# ARD1-mediated Hsp70 acetylation balances stress-induced protein refolding and degradation

**DOI:** 10.1038/ncomms12882

**Published:** 2016-10-06

**Authors:** Ji Hae Seo, Ji-Hyeon Park, Eun Ji Lee, Tam Thuy Lu Vo, Hoon Choi, Jun Yong Kim, Jae Kyung Jang, Hee-Jun Wee, Hye Shin Lee, Se Hwan Jang, Zee Yong Park, Jaeho Jeong, Kong-Joo Lee, Seung-Hyeon Seok, Jin Young Park, Bong Jin Lee, Mi-Ni Lee, Goo Taeg Oh, Kyu-Won Kim

**Affiliations:** 1 SNU-Harvard NeuroVascular Protection Research Center, College of Pharmacy, Seoul National University, Seoul 08826, Korea; 2 Department of Molecular Medicine and Biopharmaceutical Sciences, Graduate School of Convergence Science and Technology, Seoul National University, Seoul 08826, Korea; 3 School of Life Sciences, Gwangju Institute of Science & Technology, Gwangju 61005, Korea; 4 Graduate School of Pharmaceutical Sciences, College of Pharmacy, Ewha Womans University, Seoul 03760, Korea; 5 The Research Institute of Pharmaceutical Sciences, College of Pharmacy, Seoul National University, Seoul 08826, Korea; 6 Department of Life Sciences, Ewha Womans University, Seoul 03760, Korea; 7 Crop Biotechnology Institute, GreenBio Science and Technology, Seoul National University, Pyeongchang 25354, Korea

## Abstract

Heat shock protein (Hsp)70 is a molecular chaperone that maintains protein homoeostasis during cellular stress through two opposing mechanisms: protein refolding and degradation. However, the mechanisms by which Hsp70 balances these opposing functions under stress conditions remain unknown. Here, we demonstrate that Hsp70 preferentially facilitates protein refolding after stress, gradually switching to protein degradation via a mechanism dependent on ARD1-mediated Hsp70 acetylation. During the early stress response, Hsp70 is immediately acetylated by ARD1 at K77, and the acetylated Hsp70 binds to the co-chaperone Hop to allow protein refolding. Thereafter, Hsp70 is deacetylated and binds to the ubiquitin ligase protein CHIP to complete protein degradation during later stages. This switch is required for the maintenance of protein homoeostasis and ultimately rescues cells from stress-induced cell death *in vitro* and *in vivo*. Therefore, ARD1-mediated Hsp70 acetylation is a regulatory mechanism that temporally balances protein refolding/degradation in response to stress.

## Abstract (web summary)

The chaperone Hsp70 has a dual role, promoting both protein refolding and protein degradation. Seo and Park *et al*. show that Hsp70 acetylation enhances protein refolding after stress, and that subsequent deacetylation progressively promotes ubiquitin ligase binding and protein degradation.

## Graphical abstract

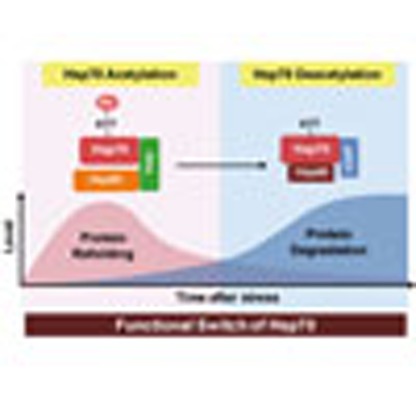

The 70 kDa heat shock proteins (Hsp70s) are a family of ubiquitously expressed intracellular proteins that are required for the maintenance of protein homoeostasis^1^,^2^,^3^,^4^. Hsp70 isoforms have been reported in *Escherichia coli* through higher organisms. In humans, a dozen Hsp70s with unique patterns of expression or subcellular localizations have been identified. Among these, Hsc70 (heat shock cognate protein, Hsp73/HSPA8) and Hsp70 (Hsp72/HSPA1A) have been extensively studied and have unique biological functions despite their high sequence homology. Hsc70 is a constitutively expressed chaperone that plays crucial roles in stabilizing protein folding under non-stress conditions^5^. In contrast, the stress-induced protein Hsp70 is highly induced in response to cellular stressors including oxidative stress, hyperthermia, hypoxia and changes in pH (ref. 6), contributing to their resistance to stress-induced cell death. Despite the distinct roles of these proteins under normal or stress conditions, the mechanisms underlying their selective regulation in different environments remain largely unknown.

Most tumour cells, which live under continuous stress conditions, express elevated levels of Hsp70 to combat these harsh conditions and suppress apoptosis. Once tumours acquire the ability to overexpress Hsp70, its expression also remains high under normal conditions^7^. This elevated Hsp70 level enables cancer cells to respond promptly to stress, in contrast to normal cells, which require time to transcribe Hsp70. However, the mechanisms responsible for the prompt or time-dependent response of Hsp70 have not been extensively studied.

The cellular response to proteotoxic stress includes protein refolding and degradation. When proteins are denatured under stress conditions, misfolded proteins can be preferentially repaired by refolding. However, if refolding fails, proteins are degraded by the ubiquitin-mediated degradation pathway^8^,^9^. The molecular chaperone Hsp70 is responsible for both protein refolding and degradation^10^,^11^,^12^, and these opposing properties of Hsp70 are closely regulated by cooperation with co-chaperones such as Hop and CHIP, which bind to Hsp70 in a competitive manner^13^. Hop and CHIP contain tetratricopeptide repeat domains that associate with the Hsp70 C terminus. Hop provides a link between Hsp70 and Hsp90 and assists in chaperone-mediated protein refolding, whereas CHIP exhibits ubiquitin ligase activity that promotes ubiquitin-mediated protein degradation. Therefore, the choice to bind with Hop or CHIP is crucial to the protein triage decision by Hsp70 of whether proteins are repaired or eliminated when they are denatured by cellular stress. However, the mechanisms by which Hsp70 chooses its binding partner and balances its opposing chaperone functions between protein refolding and degradation under stress conditions remain unknown.

Hsp70 is composed of three domains: a nucleotide-binding domain (NBD), a substrate-binding domain (SBD) and a C-terminal domain (CTD). The NBD exhibits ATPase activity that hydrolyzes ATP to ADP, and the SBD accommodates the peptides of substrate proteins. The structure of Hsp70 is highly dynamic and is dependent on ADP/ATP binding. When ADP binds to the NBD, the NBD interacts only minimally with the SBD, and peptides are able to be tightly bound to the SBD. When ATP binds to the NBD, an extensive NBD surface interacts with the SBD, and peptides can rapidly bind to and be released from the SBD. These conformational changes in Hsp70 enable the allosteric mechanisms that transfer the energetic tension from the ATP-bound NBD to the SBD^14^. Therefore, the allosteric regulation of Hsp70 is indispensable for its proper function. However, the molecular mechanisms that regulate the allostery of Hsp70 are also unknown.

The acetyltransferase ARD1 was first identified in *Saccharomyces cerevisiae*^15^. In mammals, several splice variants of ARD1 have been identified that have different biological functions^16^. Among these, human ARD1^235^ is the major isoform in humans and is involved in a wide range of biological processes including cancer development, apoptosis, differentiation and neurodegenerative disorders. ARD1 might have an important role in cell survival; however, studies of the function of ARD1 during cellular stress have yielded controversial results. For example, the depletion of ARD1 inhibited cell proliferation and induced apoptosis, indicating that ARD1 contributes to cell survival^17^. In contrast, ARD1 was required for caspase activation during apoptosis^18^ and reduced cancer cell survival through autophagy^19^. Because of these disparate results, it is uncertain whether the role of ARD1 in cellular stress is protective or apoptotic.

In this study, we elucidated the ARD1-mediated Hsp70 functional interplay between protein refolding and degradation. After stress induction, Hsp70, but not Hsc70, gradually switches its co-chaperone complex from protein refolding to protein degradation machinery to perform protein repair and elimination in sequence. This Hsp70 functional switch is regulated by ARD1-mediated acetylation. In response to cellular stress, Hsp70 is promptly acetylated at K77 by ARD1 and then gradually deacetylated at later stages. The acetylation/deacetylation state of Hsp70 regulates its co-chaperone complex, switching the chaperone functions of Hsp70 between protein refolding and degradation. In addition, the proper timing of the Hsp70 functional switch after stress induction is essential for the maintenance of protein homoeostasis and cell survival under stress conditions. Therefore, the prompt response of Hsp70 to cellular stress enables Hsp70-expressing cancer cells to be more resistant to proteotoxic stress. Thus, our study not only address how cells efficiently balance protein refolding/degradation under stress conditions but also provide a new perspective on the existence of a prompt stress response by post-translational modification that is specific to Hsp70 versus Hsc70.

## Results

### Correlation between Hsp70 acetylation and its co-chaperone

**Figure 1:**
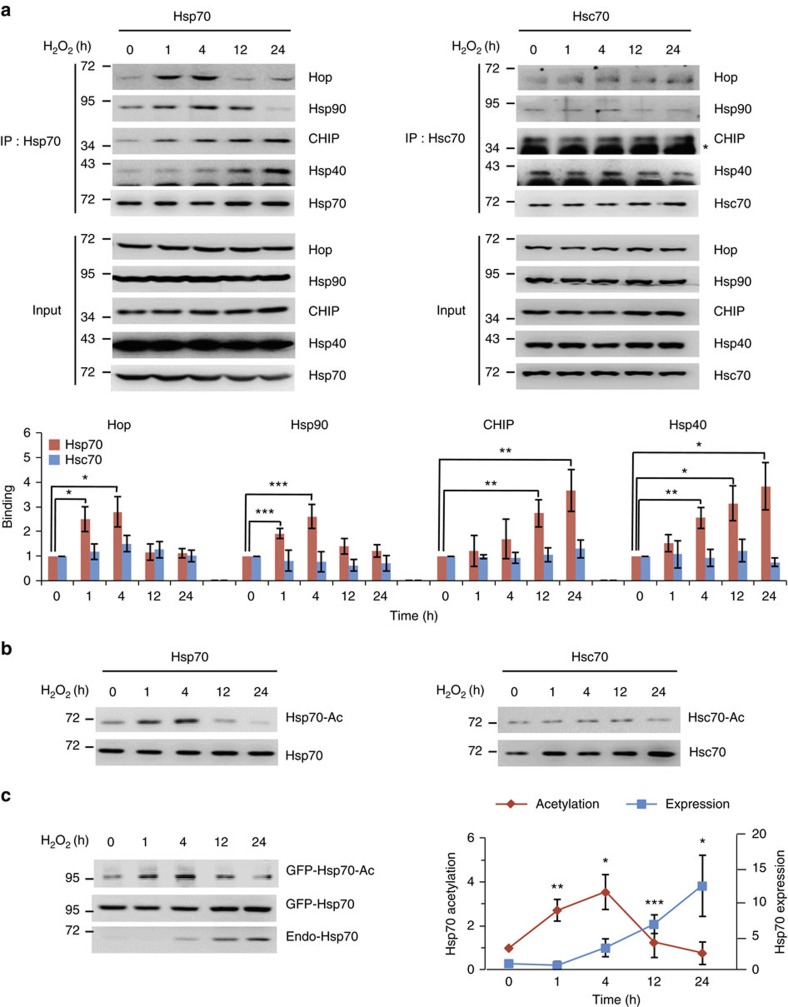
Temporal changes in co-chaperone binding to Hsp70 under stress conditions are correlated to Hsp70 acetylation. (**a**) After stress, Hsp70, but not Hsc70, gradually changes its co-chaperone complexes from protein refolding machinery (1–4 h) to degradation machinery (12–24 h). After treating HEK293T cells with 1 mM H_2_O_2_, endogenous Hsp70 and Hsc70 were precipitated, and their co-chaperone-binding partners were identified by western blotting (top). Quantification of relative co-chaperone bindings of Hsp70 and Hsc70 (bottom). The asterisk (*) indicates a background band. (**b**) Hsp70, but not Hsc70, is rapidly acetylated in response to stress. Endogenous Hsp70 and Hsc70 were precipitated from HEK293T cells treated with 1 mM H_2_O_2_, and their acetylation levels were assessed with an anti-Lys-Ac antibody. (**c**) Acetylation of Hsp70 occurs earlier than its transcriptional activation. SH-SY5Y cells stably expressing GFP-Hsp70 were treated with 1 mM H_2_O_2_, and the changes in GFP-Hsp70 acetylation and the expression of endogenous Hsp70 were compared. Error bars indicate s.d. (*n*=3). *P<0.05; **P<0.01; ***P<0.001 versus 0 h, *t* test.

To investigate the interplay of Hsp70 function between protein refolding and degradation, we first evaluated the temporal change of Hsp70 function over time after stress. Because the opposing chaperone functions of Hsp70 between protein refolding and degradation are determined by the competitive binding of co-chaperone proteins, such as Hop and CHIP, we analysed the changes in co-chaperone binding to Hsp70 over time after stress induction. Because high Hsp70 levels must be maintained during stress for the quantitative comparison of co-chaperone binding, we utilized HEK293T cells, which consistently express high levels of endogenous Hsp70 (refs 20, 21). After hydrogen peroxide treatment, the binding of Hop and Hsp90 to Hsp70, which promotes protein refolding, rapidly increased in the early phases (1–4 h) after stress induction. In contrast, the binding of CHIP and Hsp40, which promotes protein degradation, markedly increased during the later phases (12–24 h; Fig. 1a). These results suggest that Hsp70 is converted from a component of the refolding machinery during the early phases after stress to a component of the degradation machinery during the late phases. We also assessed the co-chaperone-binding changes of another Hsp70 family member, Hsc70, which is involved in normal cellular functions^22^. Unlike Hsp70, Hsc70 did not exhibit changes in co-chaperone binding after stress, suggesting the specificity of Hsp70 behaviour during stress response (Fig. 1a).

Recent studies have shown that post-translational modifications regulate various cellular functions of Hsp70 (refs 23, 24, 25, 26). In particular, investigations on stress-induced autophagy causing Hsp70 acetylation^27^ led us to hypothesize that protein acetylation might act as a switch to mediate Hsp70 function between protein refolding and degradation. To investigate this possibility, we analysed Hsp70 acetylation levels after stress. Notably, as for the co-chaperone-binding patterns associated with refolding, the acetylation level of Hsp70, but not that of Hsc70, rapidly increased during the early phases after stress and decreased during the later phases (Fig. 1b). These results suggest the possibility that the acetylation state of Hsp70 might decide between the opposing chaperone functions of Hsp70 by regulating co-chaperone binding after stress. To confirm our results under other physiological stress conditions, cellular stresses were induced by various reagents, including etoposide (a DNA-damaging reagent), 1-methyl-4-phenylpyridinium (MPP^+^, a neurotoxin), sodium chloride (hyperosmotic stress) and ethanol. Following treatment, Hsp70 acetylation rapidly increased and the co-chaperone binding changed accordingly (Supplementary Fig. 1a–d). These results indicate that the prompt stress response of Hsp70, the changes in its acetylation level, and the co-chaperone-binding pattern are conserved mechanisms shared by various types of cellular stress. In addition, we also exposed cells to brief stress instead of constant stress. Cells were briefly stimulated with hydrogen peroxide for 1 h, followed by phosphate-buffered saline (PBS) washes to remove the toxin. During the recovery period, the acetylation level and co-chaperone binding of Hsp70 gradually changed in a time-dependent manner (Supplementary Fig. 1e). These results suggest that during cellular stress, the functional switch of Hsp70 from protein refolding in the early phase to protein degradation in later phases is a serial event within the same primary response, rather than representing independent events of two parallel phases.

Because Hsp70 is inducible in many cells after stress, its transcriptional activation has been considered as a central component of Hsp70 stress response^6^. Therefore, we compared the peak period of Hsp70 acetylation with that of its transcriptional activation in SH-SY5Y cells following the stable expression of GFP-Hsp70. After stress induction, the acetylation of GFP-Hsp70 rapidly increased in the early phases (1–4 h), whereas expression of endogenous Hsp70 significantly increased in the later phases (12–24 h). These results indicate that acetylation is an immediate stress response and occurs faster than the transcriptional activation of the protein (Fig. 1c).

### ARD1 regulates the stress response of Hsp70

**Figure 2:**
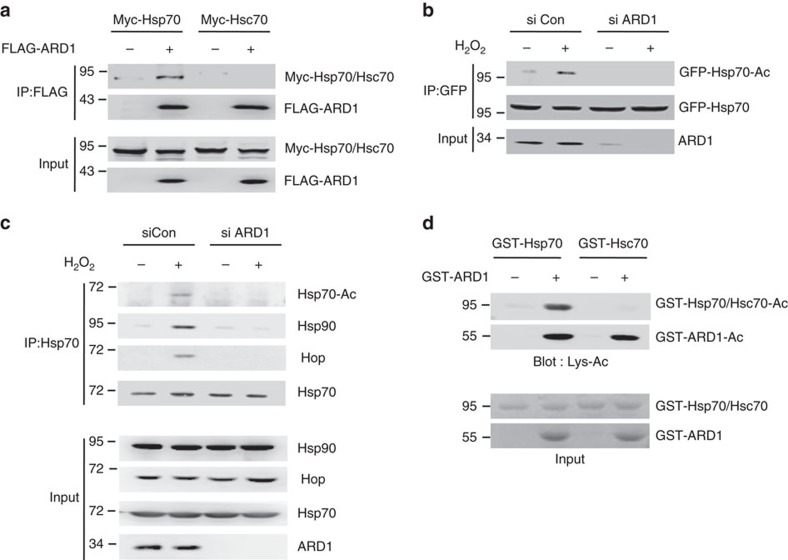
ARD1 regulates the stress response of Hsp70. (**a**) ARD1 binds to Hsp70 but not to Hsc70. FLAG-ARD1 was precipitated from HEK293T cells, and co-precipitation of Myc-Hsp70 or Myc-Hsc70 was assessed by western blotting. (**b**) ARD1 is required for stress-induced Hsp70 acetylation. SH-SY5Y cells were transfected with ARD1 siRNA. After treatment of 1 mM H_2_O_2_ for 1 h in SH-SY5Y cells, GFP-Hsp70 was precipitated, and its acetylation level was assessed with an anti-Lys-Ac antibody. (**c**) ARD1 regulates the acetylation and co-chaperone bindings of Hsp70. HEK293T cells were transfected with ARD1 siRNA. After cells were treated with 1 mM H_2_O_2_ for 1 h, endogenous Hsp70 was precipitated using an Hsp70 antibody, and then its acetylation level and co-chaperone binding were analysed by western blotting. (**d**) ARD1 acetylates Hsp70 but not Hsc70 *in vitro*. GST-ARD1, GST-Hsp70 and GST-Hsc70 recombinants were subjected to an *in vitro* acetylation assay for 1 h, and acetylation levels of recombinants were assessed by western blotting using an anti-Lys-Ac antibody.

Previously, we identified mammalian ARD1 as a lysine acetyltransferase^28^. Thereafter, we screened for ARD1-interacting proteins using affinity purification combined with mass spectrometry and observed that ARD1 bound to endogenous Hsp70 in HEK293T cells (Supplementary Fig. 2). Using co-immunoprecipitation, we observed that ARD1 bound to Hsp70 but not to Hsc70 (Fig. 2a), suggesting the involvement of ARD1 in the Hsp70-specific stress response. To determine whether ARD1 mediates an Hsp70 stress response, we assessed the acetylation level and co-chaperone binding of Hsp70 in ARD1-deficient cells. Although hydrogen peroxide rapidly increased Hsp70 acetylation in control cells, Hsp70 acetylation was significantly diminished in ARD1-deficient cells (Fig. 2b,c). Accordingly, increased binding of Hop and Hsp90 to Hsp70 at the early phase (1 h) after stress induction also did not occur in ARD1-deficient cells (Fig. 2c). These results suggest that ARD1 is critical for the prompt stress response of Hsp70. Next, an *in vitro* acetylation assay was performed to determine whether ARD1 directly acetylates Hsp70. In accordance with its selective binding pattern, recombinant GST-ARD1 directly acetylated recombinant GST-Hsp70 *in vitro*. However, GST-ARD1 did not acetylate GST-Hsc70 (Fig. 2d), demonstrating that ARD1 directly and selectively acetylates Hsp70.

### Hsp70 K77 is acetylated by ARD1

**Figure 3:**
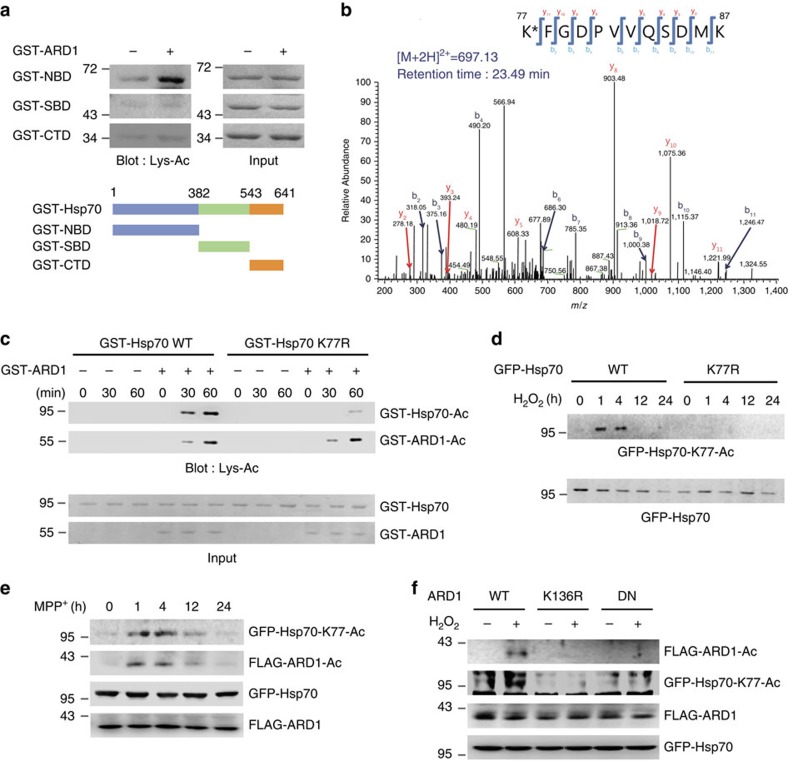
ARD1 acetylates K77 of Hsp70. (**a**) ARD1 acetylates the NBD of Hsp70 *in vitro*. Top, deletion mutants of GST-Hsp70 were subjected to *in vitro* acetylation assays with GST-ARD1. Bottom, construction of Hsp70 deletion mutants. NBD; nucleotide-binding domain, SBD; substrate-binding domain, CTD; C-terminal  domain. (**b**) K77 in GST-NBD is acetylated by ARD1 *in vitro*. The acetylation site in GST-NBD was identified by LC–MS/MS. (**c**) ARD1 acetylated K77 of Hsp70 *in vitro*. GST-Hsp70 WT and GST-Hsp70 K77R recombinants were subjected to *in vitro* acetylation assays with or without GST-ARD1 recombinant. Acetylation levels of GST-Hsp70 recombinants were determined using an anti-Lys-Ac antibody. (**d**) K77 acetylation of Hsp70 was increased by cellular stress. After treatment of HEK293T cells with 1 mM H_2_O_2_, K77 acetylation of GFP-Hsp70 was assessed by western blotting using an anti-Hsp70-K77-Ac antibody. (**e**) ARD1 and Hsp70 are acetylated by cellular stress. After treatment with 1 mM MPP^+^ in SH-SY5Y cells, K77 acetylation of GFP-Hsp70 was determined by western blotting using an anti-Hsp70-K77-Ac antibody. Acetylation of FLAG-ARD1 was assessed by immunoprecipitation using an anti-Lys-Ac antibody. (**f**) ARD1 autoacetylation at K136 is required for stress-induced Hsp70 acetylation. HEK293T cells expressing the indicated ARD1 plasmids were treated with 1 mM H_2_O_2_. Acetylation levels of FLAG-ARD1 and GFP-Hsp70 were assessed by immunoprecipitation using an anti-Lys-Ac antibody and western blotting using an anti-Hsp70-K77-Ac antibody, respectively. DN; dominant-negative mutant.

To identify the target site of ARD1-mediated Hsp70 acetylation, we constructed Hsp70 deletion mutants and subjected them to an *in vitro* acetylation assay. The NBD of Hsp70 was acetylated by ARD1 *in vitro* (Fig. 3a). To identify the acetylation site, acetylated GST-NBD was digested into peptides and then subjected to micro-liquid chromatography-tandem mass spectrometry (LC–MS/MS), which showed that the peptide containing the Lys 77 residue (K77) was acetylated. The peptide identity and the acetylation residue position were confirmed via the MS/MS spectra (Fig. 3b). To establish the causality of this relationship, we mutated the K77 of recombinant Hsp70 to an Arg 77 residue (K77R) and performed an *in vitro* acetylation assay. Although wild-type GST-Hsp70 was acetylated by ARD1 in a time-dependent manner, the K77R mutation significantly decreased the ARD1-mediated acetylation of GST-Hsp70, indicating that K77 is the main target site of ARD1-mediated Hsp70 acetylation (Fig. 3c). Although K77 was the only acetylated site discovered using LC–MS/MS, the K77R mutation did not completely diminish Hsp70 acetylation, suggesting the existence of other, minor acetylation sites. To exclude their effects and focus on Hsp70 K77 acetylation, we constructed an antibody specific for this modification (Hsp70-K77-Ac) (Supplementary Fig. 3).

Next, to examine whether K77 acetylation of Hsp70 was upregulated by cellular stress, we measured the change in Hsp70 K77 acetylation level in cells after stress induction. Using the Hsp70-K77-Ac specific antibody, we determined that Hsp70 K77 acetylation rapidly increased following hydrogen peroxide treatment in HEK293T cells (Fig. 3d). Similar results were also observed in MPP^+^-treated SH-SY5Y cells (Fig. 3e), confirming the stress-induced K77 acetylation of Hsp70. Notably, we observed that ARD1 was also acetylated in a similar pattern under the same conditions (Fig. 3e). These results indicate the existence of an acetylation cascade that sequentially activates ARD1 and Hsp70 following stress. Previously, we reported that ARD1 is autoacetylated at K136, and this autoacetylation is a critical step to stimulate the catalytic activity of ARD1 (ref. 29). To determine whether the autoacetylation of ARD1 is required for the Hsp70-mediated stress response, we used K136R and dominant-negative mutants of ARD1 that inhibit its autoacetylation activity. These two mutants eliminated hydrogen peroxide-stimulated ARD1 acetylation and completely blocked stress-induced Hsp70 K77 acetylation (Fig. 3f), indicating that in response to cellular stress, ARD1 is activated through autoacetylation, and that this step represents the upstream signal for stress-induced Hsp70 acetylation.

### K77 acetylation enhances Hsp70 chaperone activity

**Figure 4:**
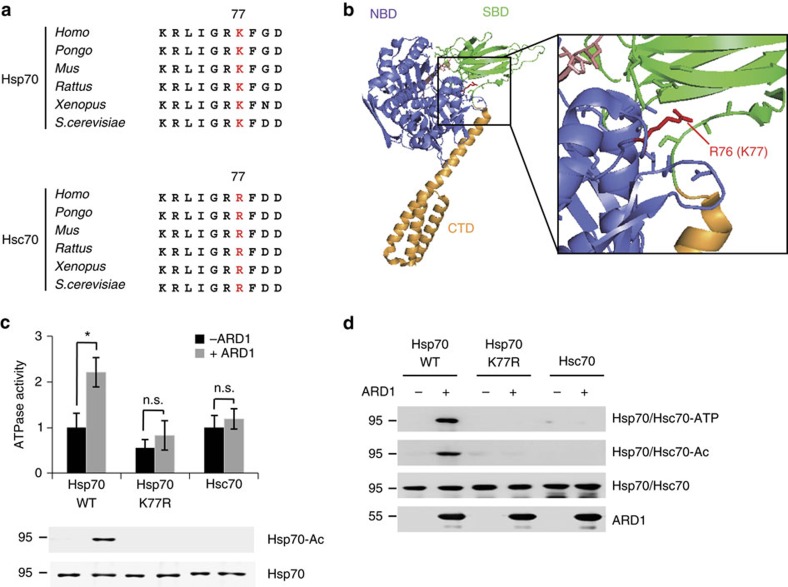
K77 acetylation enhances the chaperone activity of Hsp70. (**a**) The K77 residue is conserved in Hsp70 but not in Hsc70. Sequence alignment of Hsp70 (top) and Hsc70 (bottom) in various species. (**b**) The acetylation site is located in the NBD-SBD interface of Hsp70-ATP. The position of the Hsp70 acetylation site is indicated in red within the structure of DnaK-ATP (PDB: 4JNE), the *E. coli* homologue of human Hsp70-ATP. (**c**,**d**) K77 acetylation upregulates the ATPase cycle of Hsp70. GST-Hsp70 and GST-Hsc70 recombinant proteins were subjected to *in vitro* acetylation assay with or without ARD1. For ATPase activity assay, recombinant proteins were incubated with ATP for 1 h, then ATP hydrolysis was measured (**c**). For ATP-binding assay, recombinant proteins were pulled-down with ATP-agarose bead, and ATP-bound recombinant proteins were analysed by western blotting (**d**). Error bars indicate s.d. (*n*=3). *P<0.05, *t* test.

To determine whether ARD1-mediated acetylation site at Hsp70 K77 is conserved in various species, we compared the amino acid sequences across species. Sequence alignment revealed that this site is highly conserved from yeast to humans, whereas it is not conserved in Hsc70. Hsc70 contains an R77 residue instead of K77, which might explain why Hsc70 is not acetylated after stress and also supports the specialized function of Hsp70 during stress response (Fig. 4a).

Although Hsp70 has an ATPase enzyme activity, the protein modification that regulates this activity is unknown. Therefore, we next evaluated whether Hsp70 K77 acetylation affects its enzymatic activity. Because Hsp70 is allosterically activated by an ADP/ATP binding-induced conformational change, we first predicted the location of K77 in ADP-bound and ATP-bound structures. As the crystal structure of human Hsp70 is unknown, we used the crystal structure of DnaK, the Hsp70 homologue of *E. coli*^30^. In DnaK, the R76 residue corresponds to K77 of human Hsp70 (Supplementary Fig. 4a) and is located in the interface between the NBD and SBD of the ATP-bound form (Fig. 4b; Supplementary Fig. 4b). Because the interaction of NBD with SBD is an important event for the allosteric activation of Hsp70 (refs 14, 30, 31), the putative location of K77 suggests an important role for K77 acetylation in regulating Hsp70 chaperone activity. To determine whether K77 acetylation indeed regulates the ATPase cycle of Hsp70, we assessed the ATPase activity and ATP-binding affinity of Hsp70 using wild-type and K77R mutant GST-Hsp70 recombinants. These functions of wild-type Hsp70 were significantly enhanced when Hsp70 was acetylated by ARD1 *in vitro* (Fig. 4c,d), but exhibited no ARD1-induced change in the K77R mutant Hsp70 and Hsc70 recombinants, consistent with their acetylation state (Fig. 4c,d). Together, these results suggest that ARD1-mediated K77 acetylation enhances the chaperone activity of Hsp70.

### Acetylation state of Hsp70 determines its chaperone function

**Figure 5:**
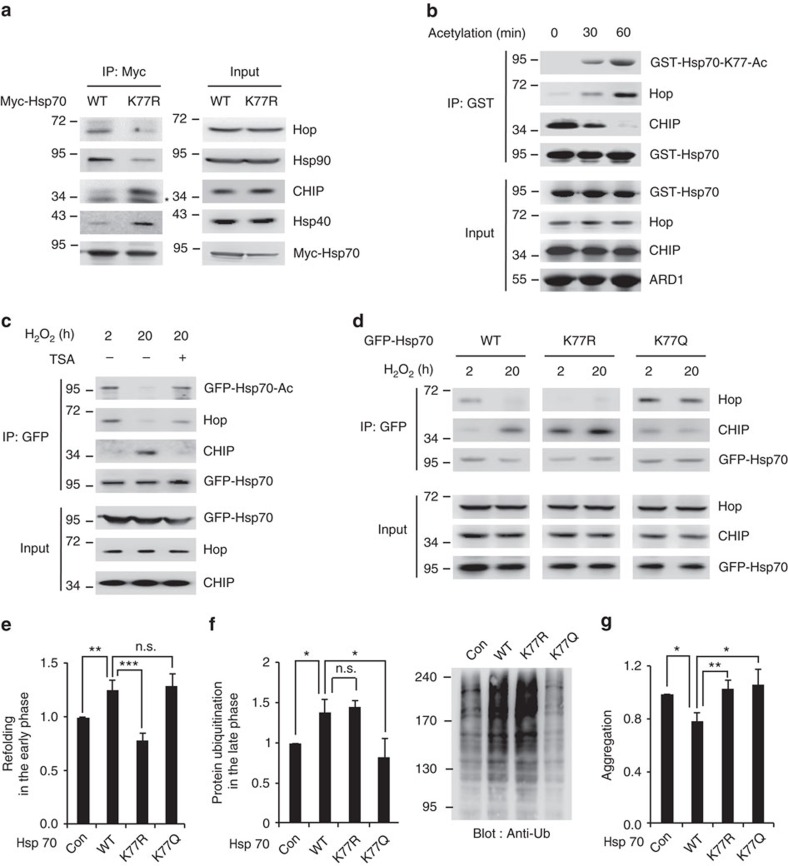
K77 acetylation temporally balances the chaperone functions of Hsp70 by regulating co-chaperone binding. (**a**) K77 acetylation controls the co-chaperone-binding complexes of Hsp70. Myc-Hsp70 WT and K77R were precipitated from HEK293T cells, and the co-precipitated co-chaperones were assessed. The asterisk (*) indicates a background band. (**b**) K77 acetylation of Hsp70 regulates the competitive binding of Hop and CHIP *in vitro*. GST-Hsp70 was acetylated by GST-ARD1 and subjected to *in vitro*-binding assay. Co-precipitated Hop or CHIP with GST-Hsp70 was assessed by western blotting. (**c**) The acetylation state of Hsp70 determines Hop and CHIP binding after stress. HEK293T cells expressing GFP-Hsp70 were treated with 1 mM H_2_O_2_ for the indicated times with or without TSA treatment (4 h). GFP-Hsp70 was precipitated using an anti-GFP antibody, and its acetylation and co-chaperone bindings were assessed. (**d**) During stress, Hsp70 switches its co-chaperone binding depending on its acetylation/deacetylation state. HEK293T cells were treated with 1 mM H_2_O_2_ for the indicated times. Temporal changes in co-chaperone binding of GFP-Hsp70 were determined by immunoprecipitation. (**e**) Acetylated Hsp70 encourages protein refolding in the early phase after stress. The K77R mutation of Hsp70-impaired protein refolding in the early phase after stress. HEK293T cells transfected with pRSV-luciferase and the indicated Hsp70 plasmids were treated with 0.3 mM H_2_O_2_ for 30 min. After 2 h of recovery, firefly luciferase activity was measured. (**f**) Deacetylated Hsp70 controls protein ubiquitination in the late phase after stress. K77Q mutation of Hsp70 impaired protein degradation in the late phase after stress. To estimate the amount of protein degradation, HEK293T cells were treated with 1 mM H_2_O_2_ (20 h) and total protein ubiquitination was measured by western blotting (right) and quantified (left). (**g**) The switch between Hsp70 acetylation/deacetylation is required to maintain protein homoeostasis after stress. Stress-induced protein aggregation was enhanced by both the K77R and K77Q mutations. HEK293T cells expressing the indicated Hsp70 plasmids were treated with 1 mM H_2_O_2_ (24 h). Protein aggregation was analysed by protein aggregation detection dye. Error bars indicate s.d. (*n*=3). *P <0.05; **P <0.01; ***P <0.005, *t* test. n.s.; not significant.

Hsp70 conformational change induced by ADP/ATP binding is also related to its co-chaperone affinity, which decides whether Hsp70 functions as a chaperone for protein refolding or degradation^32^,^33^. Therefore, we next determined whether the composition of Hsp70 co-chaperone complex is regulated by K77 acetylation. We transfected wild-type or K77R mutant Hsp70 into HEK293T cells and then compared their binding affinities with co-chaperones. Compared with wild-type Hsp70, the K77R mutation weakened the binding of Hsp70 to Hop and Hsp90 and enhanced its affinity for CHIP and Hsp40 (Fig. 5a). To clarify the direct effects of Hsp70 acetylation on the competitive binding of Hop and CHIP to Hsp70, we performed an *in vitro* binding assay. After the Hsp70 recombinant was acetylated by ARD1 *in vitro*, Hop and CHIP recombinants were subjected to an *in vitro* competitive-binding assay. Acetylated Hsp70 preferred to associate with Hop rather than CHIP, whereas nonacetylated Hsp70 bound to CHIP instead of Hop (Fig. 5b). These results suggest that the K77 acetylation status of Hsp70 determines its chaperone function between refolding and degradation by directly regulating Hop and CHIP binding. To further elucidate whether the co-chaperone complex is altered by the acetylation/deacetylation state of Hsp70 under stress conditions, we treated cells with the histone deacetylase (HDAC) inhibitor trichostatin A (TSA) and measured the changes in Hsp70 acetylation levels and co-chaperone binding. Consistent with the data shown in Fig. 1a,b, depending on the acetylation state, Hsp70 bound to Hop during the early phase (2 h) and to CHIP during the late phase (20 h) after stress induction (Fig. 5c). However, after TSA treatment for 4 h at the late phase (20 h), Hsp70 acetylation was recovered, and Hsp70 consequently changed its binding partner from CHIP to Hop (Fig. 5c). In addition, we also observed that HDAC4 bound to and deacetylated Hsp70 (Supplementary Fig. 5a and b). In HDAC4-overexpressing cells, Hsp70 acetylation levels were reduced and Hsp70 co-chaperone binding was accordingly altered (Supplementary Fig. 5c). In HDAC4-deficient cells, Hsp70 was not deacetylated in the late phase (20 h) and switch of co-chaperone bindings was also disappeared, indicating that HDAC4 is a relevant deacetylase for Hsp70 (Supplementary Fig. 5d). These data also confirm that the co-chaperone binding of Hsp70 can switch and is dependent on its acetylation/deacetylation state.

To further confirm our results, we transfected cells with the deacetylation-mimic K77R and acetylation-mimic K77Q Hsp70 mutants and checked the temporal switching of their co-chaperone binding from early phase (2 h) to late phase (20 h) response. Although wild-type Hsp70 temporally switched its co-chaperone-binding partner from Hop in the early phase (2 h) to CHIP in the later phase (20 h), the K77R and K77Q Hsp70 mutants could not convert their binding partners under the same condition (Fig. 5d; Supplementary Fig. 6). Throughout the period of cellular stress, the K77R and K77Q mutants consistently bound to CHIP and Hop, respectively (Fig. 5d; Supplementary Fig. 6). These results indicate that the conversion of acetylation/deacetylation state of Hsp70 acts as a key switch that temporally balances the Hsp70 co-chaperone complex between the refolding machinery and the degradation machinery under stress conditions.

Next, to determine whether this temporal switch of co-chaperone binding to Hsp70 between early and late phases after stress is necessary for the maintenance of protein homoeostasis under stress conditions, we measured protein refolding at the early phase and degradation at the late phase using the Hsp70 K77R and K77Q mutants. Consistent with the defects in co-chaperone binding, the K77R mutation of Hsp70 impaired its capacity for protein refolding during the early phase after stress (Fig. 5e; Supplementary Fig. 7), whereas it exhibited normal protein degradation activity in the late phase (Fig. 5f; Supplementary Fig. 8). In contrast, the K77Q mutant exhibited normal protein refolding activity during the early phase (Fig. 5e; Supplementary Fig. 7), but exhibited defects in ubiquitin-mediated protein degradation during the later phase (Fig. 5f; Supplementary Fig. 8). Consequently, the final accumulation of protein aggregates caused by the failure of protein refolding or degradation was elevated by both mutations after stress (Fig. 5g; Supplementary Fig. 9). These results indicate that the Hsp70 switch from facilitating protein refolding to facilitating degradation at the proper time under stress conditions is essential for the maintenance of protein homoeostasis.

### Hsp70 acetylation protects cells against stress

**Figure 6:**
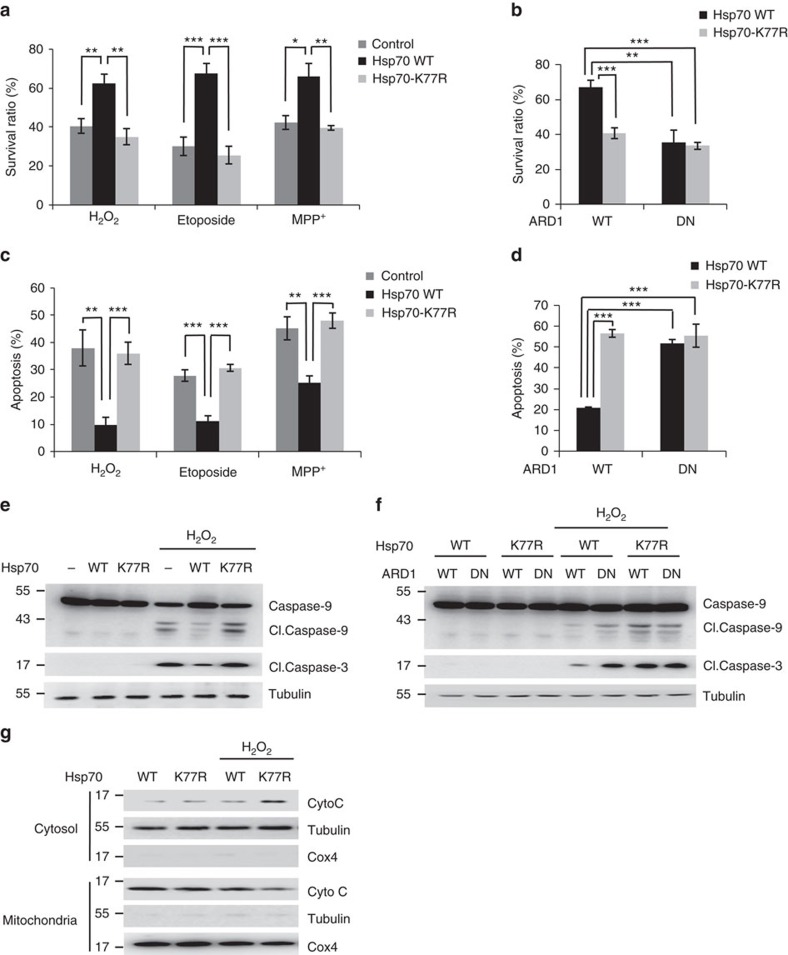
ARD1-mediated Hsp70 acetylation protects cells against various types of cellular stress. (**a**) Hsp70 acetylation supports cell survival against various types of cellular stress. SH-SY5Y cells expressing GFP-Hsp70 WT and K77R were stimulated with 1 mM H_2_O_2_ for 24 h, 100 mM etoposide for 24 h and 1 mM MPP^+^ for 48 h, and the survival ratios were measured. (**b**) ARD1 promotes cell survival by Hsp70 acetylation after stress. After 1 mM H_2_O_2_ treatment for 24 h, the survival ratios of SH-SY5Y cells expressing GFP-Hsp70 and FLAG-ARD1 were analysed. (**c**) Hsp70 acetylation inhibits stress-induced apoptosis. The ratio of apoptotic cells was measured in SH-SY5Y cells treated with 0.3 mM H_2_O_2_ for 24 h, 100 mM etoposide for 24 h and 1 mM MPP^+^ for 48 h. (**d**) ARD1 inhibits apoptosis through Hsp70 acetylation. SH-SY5Y cells were treated with 0.3 mM H_2_O_2_ for 24 h, and the ratio of apoptotic cells was measured. (**e**) Hsp70 acetylation inhibits stress-induced activation of caspase-3 and -9. After treatment with 0.3 mM H_2_O_2_ for 24 h, the cleavage (Cl.) of caspase-3 and -9 was detected by western blotting. (**f**) ARD1 inhibits the activation of caspase-3 and -9 through Hsp70 acetylation. After treatment with 0.3 mM H_2_O_2_ for 24 h, the cleavage (Cl.) of caspase-3 and -9 was detected in SH-SY5Y cells co-expressing GFP-Hsp70 and FLAG-ARD1. (**g**) Hsp70 acetylation inhibits cytochrome *c* release from mitochondria. After treating 0.3 mM H_2_O_2_ with HEK293T cells expressing GFP-Hsp70 WT and K77R, mitochondrial and cytosolic proteins were fractionated and applied to western blotting. Error bars indicate s.d. (*n*=3). *P<0.05; **P<0.01; ***P<0.001, *t* test.

Stress-induced disruption of protein homoeostasis eventually results in cell dysfunction or cell death, and Hsp70 plays a protective role during these processes^34^,^35^. Therefore, we investigated the effect of Hsp70 acetylation on cell survival under various types of cellular stress using stable cell lines expressing wild-type and mutant Hsp70 and ARD1. Wild-type Hsp70-protected cells against various cellular stresses, but this protective effect was eliminated by the K77R mutation (Fig. 6a). In addition, the protective effect was abolished by co-expression with dominant-negative mutant ARD1 (Fig. 6b), indicating that ARD1 supports cell survival during cellular stress through Hsp70 acetylation. Similarly, the K77Q mutant also impaired the protective effect of wild-type Hsp70 despite its normal ATPase activity (Supplementary Fig. 10). These results suggest that in Hsp70-mediated stress response, although Hsp70 exhibits normal ATPase activity throughout, the temporal switch of chaperone functions between protein refolding and degradation is indispensable to protect cells under stress conditions.

Next, we also examined the effect of Hsp70 acetylation on stress-induced apoptosis. As expected, wild-type Hsp70 decreased the apoptotic cell number after the induction of various stresses, whereas the Hsp70 K77R mutant showed no effect (Fig. 6c). The anti-apoptotic effect of wild-type Hsp70 was eliminated in dominant-negative mutant ARD1-expressing cells (Fig. 6d). These results were confirmed using apoptotic markers including cleavage of caspase-9 and -3 and cytochrome *c* release from mitochondria. Wild-type Hsp70 inhibited the stress-induced cleavage of caspase-9 and -3, however, this protective effect was abolished by K77R mutation of Hsp70 (Fig. 6e). Similarly, co-expression of dominant-negative mutant ARD1 also reduced the protective effect of wild-type Hsp70 (Fig. 6f). We further observed that stress-induced cytochrome *c* release from mitochondria was increased by K77R mutation of Hsp70 (Fig. 6g). These results indicates that ARD1-mediated Hsp70 acetylation has an anti-apoptotic effect during cellular stress.

The protective effects of Hsp70 acetylation were also confirmed *in vivo* using zebrafish expressing Hsp70 with or without ARD1. To induce lethality, zebrafish were incubated with hydrogen peroxide and survival ratios were measured. Consistent with the *in vitro* data shown in Fig. 6a, the K77 mutation in Hsp70 reduced the protective effect of HSP70 against hydrogen peroxide-induced oxidative stress (Supplementary Fig. 11a). Furthermore, the protective effect of Hsp70 K77 acetylation was blocked by co-expression of the dominant-negative mutant ARD1 (Supplementary Fig. 11b). In addition, we treated zebrafish with 1-methyl-4-phenyl-1,2,3,6-tetrahydropyridine (MPTP) and measured the loss of dopaminergic neurons. Compared with zebrafish expressing wild-type Hsp70, zebrafish expressing the Hsp70 K77R mutant exhibited increased dopaminergic neuron loss (Supplementary Fig. 11c,d). Functionally, the K77R mutation markedly decreased zebrafish locomotor activity compared with that in wild-type Hsp70 (Supplementary Fig. 11e), and expression of the dominant-negative mutant ARD1 eliminated the protective effects of wild-type Hsp70 (Supplementary Fig. 11f). These results confirm the protective role of ARD1-mediated Hsp70 acetylation *in vivo*.

## Discussion

**Figure 7:**
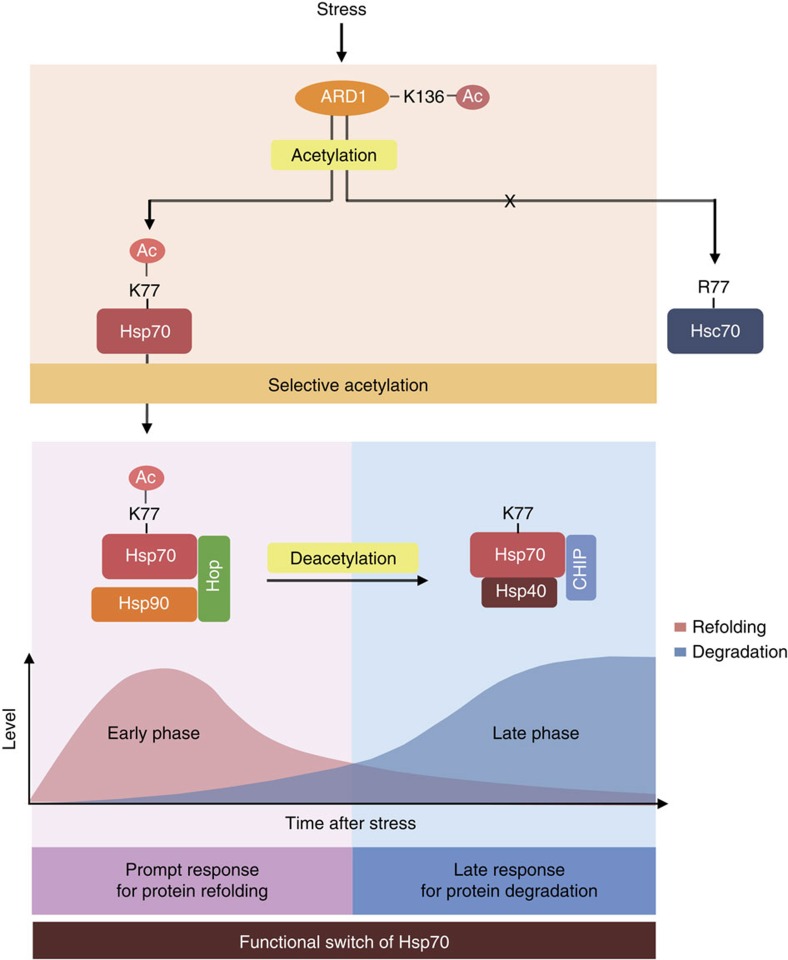
Schematics for functional switch of Hsp70 regulated by ARD1-mediated acetylation. After stress, ARD1 and Hsp70 are sequentially activated by an acetylation cascade. ARD1 selectively acetylates Hsp70 but not Hsc70. In the early phase after stress, Hsp70 is rapidly activated by acetylation to support protein refolding. In the late phase, deacetylated Hsp70 contributes to protein degradation.

This study presents a molecular mechanism that temporally balances protein refolding and degradation mediated by Hsp70 under stress conditions. We propose a model of Hsp70 functional interplay between protein refolding and degradation during cellular stress (Fig. 7). In response to stress, Hsp70 is rapidly activated by acetylation to preferentially encourage refolding of denatured proteins. However, when Hsp70 reaches the limit of protein repair, its acetylation is gradually decreased thus converting its function from misfolded protein repair to misfolded protein degradation.

The chaperone-mediated protein triage decision between protein refolding and degradation is modulated by the co-chaperone complex. However, how the different co-chaperones are associated with and influence the Hsp70 decision was unknown. The present study investigated Hsp70 acetylation as a potential switch for its mode of action. We observed that the Hsp70 acetylation state decides between protein refolding and degradation by controlling the competitive binding of Hop and CHIP. The possibility that protein modification affects co-chaperone binding was also recently suggested in previous reports. Muller *et al*. and Assimon *et al*. showed that phosphorylation of the C termini of Hsp70 and Hsp90 enhanced their binding to Hop^25^,^36^. In addition, Röhl *et al*.^37^ reported that phosphorylation of Hop decreased its binding affinity to Hsp70 *in vitro*. It is possible that these modifications are correlated with K77 acetylation of Hsp70 under stress conditions. For example, Hsp70 may be phosphorylated in the early phase after stress and synergize the effect of acetylation of Hsp70 to promote Hop binding. In contrast, phosphorylation of Hop may be stimulated during the late phase after stress to become separated from deacetylated Hsp70. Therefore, further studies are needed to investigate how these post-translational modifications are sequentially stimulated over time after stress induction and how they interact with each other to regulate Hsp70 function.

In this study, Hsp70 acetylation site is located in the NBD, which does not directly interact with Hop and CHIP. Therefore, this modification may affect C-terminal binding of these proteins through an Hsp70 conformational change. Hsp70 is a representative enzyme regulated by allosteric mechanisms. Without direct interactions, distal domains can be allosterically modulated by co-factors such as nucleotides, ions, disulfide bonds and post-translational modifications^38^. Several studies have shown that phosphorylation-induced allosteric conformational changes modulate protein activity^39^,^40^,^41^. Thus, K77 acetylation in NBD may induce allosteric conformational changes in SBD and CTD, resulting in changes in co-chaperone-binding preference. In addition, K77 is located at a significant position for NBD-SBD interdomain contacts, which adds another interesting possibility that K77 acetylation at the NBD-SBD interface may modulates the Hsp70 conformational change. Although detailed investigations are needed to elucidate the exact mechanisms, our results provide insight into the allosteric mechanism of Hsp70 regulation by post-translational modifications.

Hsp70 is induced by cellular stress^42^. Therefore, most Hsp70 studies have focused on its transcription. However, transcriptional activation is not sufficient to explain the enzymatic characteristics of Hsp70 as an ATPase. Furthermore, Hsp70 is already highly expressed in many cells including cancer cells. Thus, we speculated the existence of a prompt Hsp70 stress response that could react more immediately than allowed by transcriptional activation and regulate the enzymatic activity of Hsp70 after cellular stress. Post-translational modification is a representative cellular switch that dynamically controls the cellular functions of various proteins. Relative to transcriptional activation, post-translational modification is a more efficient, rapid response to changes in the cellular environment and can reversibly turn on/off enzyme activity. Indeed, recent studies have suggested that such modifications regulate the diverse Hsp70 cellular functions involved in cell cycle regulation, cancer proliferation and autophagy^23^,^24^,^25^,^26^,^27^. Consistent with these results, our study revealed that Hsp70 could respond immediately to cellular stress through its acetylation. In response to stress, Hsp70 acetylation occurred more rapidly than transcriptional activation (Fig. 1c), indicating the existence of a novel, prompt Hsp70 stress response regulated by post-translational modification.

This prompt Hsp70 stress response ultimately contributes to the effective disposal of stress-induced denatured proteins. Specifically, acetylated Hsp70 prioritizes protein repair in the early phase after stress. Then, the remaining unrepaired proteins are eliminated by deacetylated Hsp70 during the late phase. This temporal distribution of protein refolding/degradation is an effective measure to overcome cellular stress. In cells that rarely express Hsp70, Hsp70-mediated protein refolding in the early phase might be weak; therefore, denatured proteins can be more strongly regulated by the protein degradation pathway in the late phase after sufficient Hsp70 is synthesized. We suggest that the existence of a prompt Hsp70 stress response might be one reason why Hsp70-expressing cells are more resistant to proteotoxic stress. For this reason, many cells including cancer cell might maintain elevated levels of Hsp70 under normal conditions as a preventive measure to allow immediate response to unexpected cellular stress.

ARD1 was originally identified in yeast as an NH_2_-terminal acetyltransferase that acetylates the NH_2_-terminal amino acid of newly synthesized proteins. In mammalian cells, we found that ARD1 exhibits lysine acetyltransferase activity that acetylates the internal lysine residues of proteins^28^. Thereafter, diverse ARD1 substrates were identified and various cellular functions of ARD1 in angiogenesis, cancer proliferation, cell cycle regulation, apoptosis, development and differentiation were reported^43^,^44^. In the present study, we identified Hsp70 as a new substrate of ARD1 and also suggested a novel role for ARD1 in the regulation of protein homoeostasis. Through acetylation of Hsp70 K77, ARD1 enhanced the ATPase activity and regulated the chaperone function of Hsp70 required for cell protection against stress conditions. In addition, similar to other enzymes that regulate post-translational modification, ARD1 can activate itself through autoacetylation^29^. Notably, ARD1 was rapidly autoacetylated in response to stress, and this step was indispensable for Hsp70 acetylation. These results suggest the presence of an acetylation cascade for sensing cellular stress and directing the downstream response.

Hsp70 acetylation was also observed in other studies. Treatment with HDAC inhibitors increased intracellular Hsp70 and Hsp90 acetylation levels^45^. Furthermore, Hsp70 K159 was acetylated and deacetylated by p300 and HDAC6, respectively^27^. In this study, we found that HDAC4 deacetylates the K77 acetylation of Hsp70 and changes its co-chaperone binding. On the basis of the acetylation/deacetylation state, we assume that Hsp70 may bind more strongly to ARD1 in the early phase after stress, then Hsp70 may gradually convert its binding from ARD1 to HDAC4 over time. Therefore, further studies are needed to investigate the component proportion ratio of HDAC4 and ARD1 in the Hsp70 complex and its modulation over time after cellular stress. In addition, we did not examine all HDAC family members in this study, therefore, the involvement of other HDACs in K77 deacetylation of Hsp70 is also needed to be examined in further studies.

In this study, we used two Hsp70 mutants, K77R and K77Q, which mimic deacetylation and acetylation, respectively, to investigate the effect of ARD1-mediated Hsp70 acetylation. The K77R mutation significantly downregulated the ATPase activity of Hsp70, whereas K77Q showed no change (Supplementary Fig. 10b). However, both mutants failed to convert their co-chaperone binding according to early or late phase response, resulting in reduced cell survival under stress conditions (Fig. 5d; Supplementary Fig. 10a). These results indicate that Hsp70 acetylation/deacetylation does not simply represent the active/inactive state of Hsp70, but rather that the dynamic conversion of Hsp70 between acetylation and deacetylation states is critical for its proper cellular function.

Although Hsp70 and Hsc70 are indiscriminately considered in most studies, because of their highly similar roles and sequence homologies, their differences warrant further investigation. For example, Hsp70 promotes cancer growth and survival, whereas Hsc70 is more relevant for normal cellular growth. Therefore, the selective inhibition of Hsp70 is a promising approach for the development of selective cancer therapies that do not disturb the normal cellular function of Hsc70. However, the high similarity between these proteins makes it difficult to develop a selective inhibitor for Hsp70.

In the present study, the prompt stress response mediated by ARD1 acetylation was specific for Hsp70 but not for Hsc70 (Fig. 1). The distinguishable stress responses of Hsp70 and Hsc70 might be explained by their differing amino acids at residue 77, where instead of lysine (K), Hsc70 contains arginine (R), which cannot be acetylated (Fig. 4). Therefore, we hypothesize that ARD1-mediated selective acetylation of Hsp70 might contribute to the specialized functions of Hsp70 and Hsc70 in different cellular environments.

In conclusion, our study identified a selective Hsp70 regulatory mechanism and offers a promising approach for the development of a specific inhibitor of Hsp70 that does not target the normal cellular functions of Hsc70. Regulation of Hsp70 K77 acetylation might be helpful in treating various diseases involving Hsp70 including cancer development, inflammatory diseases and neurodegeneration^46^.

## Methods

### Animals

Wild-type AB/TU zebrafish embryos were maintained and staged according to standard laboratory protocols. Experiments were performed in accordance with protocols approved by the Institutional Animal Care and Use Committee at Seoul National University.

### Cell culture and stimulation

SH-SY5Y and HEK293T cells were obtained from ATCC. Cells were grown in DMEM supplemented with 10% fetal bovine serum and 1% penicillin/streptomycin in 5% CO_2_ humidified atmosphere at 37 °C. To induce cellular stress, cells were treated with 1 mM H_2_O_2_, 100 μM etoposide, 1 mM 1-methyl-4-phenylpyridinium (MPP^+^), 100 mM NaCl or 150 mM EtOH.

### siRNA and plasmid construction

The siRNA sequence targeting human ARD1 corresponds to CUGACUGCGCUUCACGAU (Dharmacon) as previously described^29^. The siRNA targeting human HDAC4 (#7595) was purchased from Cell Signaling Technology. Full-length of cDNAs for human Hsp70 (Genbank: NM_005355.5), human Hsc70 (Genbank: NM_00697.5) and human ARD1(Genbank: NM_003491.3) were obtained from PCR and subcloned into pCDNA3.1 (FLAG-ARD1), pCS2^+^ (Myc-ARD1, Myc-Hsp70 and Myc-Hsc70) and pEGFP-C3 (GFP-Hsp70) vectors for cellular expression or pGEX-4T (GST-ARD1, GST-Hsp70 and GST-Hsc70) vectors for the bacterial induction of the recombinant protein. A deletion mutants of GST-Hsp70 were constructed from pGEX-4T-Hsp70 plasmid. cDNAs of Hsp70 corresponding to 1–382 aa, 383–543 aa and 544–641 aa were amplified by PCR and inserted into pGEX-4T-1. For construction of stable cell lines, cDNAs of Hsp70 and ARD1 were co-inserted into pIRES vector purchased from Clontech. For luciferase refolding assay, pRSV-luciferase vector was purchased from addgene^47^. Point mutations in Hsp70 (K77R and K77Q) and ARD1 (K136R and DN: R82A/Y122F) were generated using the Muta-Direct Site Directed Mutagenesis kit (Intron) according to the manufacturer's instructions and the following primers and its reverse-complement for each point mutation (mutated based in lower case bold):

Hsp70 K77R: CGGCTGATTGGCCGCA**g**GTTCGGCGACCCGGTG

Hsp70 K77Q: GCGGCTGATTGGCCGC**c**AGTTCGGCGACCCGGT

ARD1 K136R: AGTGAAGTGGAGCCCA**g**ATACTATGCAGATGGG

ARD1 R82A: TGTGAAGCGTTCCCAC**gc**GCGCCTCGGTCTGGCT

ARD1 Y122F: GCCGCCCTGCACCTCT**t**TTCCAACACCCTCAAC

### Transfection

Transfection was carried out as described previously^48^. We performed HEK293T cell transfection with polyethyleneimine (PEI) at a ratio of 4:1 (μl PEI/μg plasmid DAN) in basal media overnight followed by change of media. siRNA targeting ARD1 was transfected with Oligofectamin reagent (Invitrogen) according to the manufactor's instruction.

For the establishment of stable cells, pEGFP-C3-Hsp70 and pIRES-GFP-Hsp70-FLAG-ARD1 plasmids were transfected into SH-SY5Y cells using PolyFect reagent (Qiagen) according to the manufactor's instruction. Transfected cells were maintained in complete DMEM with G418 (500 μg ml^−1^). After several days, the surviving colonies were selected and amplified. Expression of GFP-Hsp70 and FLAG-ARD1 was quantified by western blot.

### Antibodies

For a custom anti-rabbit Hsp70-K77-Ac antibody, rabbits were immunized with synthetic peptide-bearing acetylated human Hsp70 (Ab_72–81_:RLIGRK-acFGDP) following standard procedures (Peptron and Abfrontier). This antibody was purified with antigen-specific affinity chromatography and blocked by non-acetylated synthetic peptide (Ab_72–81_:RLIGRKFGDP) before use. Anti-Hsp70 antibody (C92F3A-5, ADI-SPA-810, 1:3,000) and anti-Hsc70 antibody (1B5, ADI-SPA-815, 1:3,000) were purchased from Enzo Lifescience. Anti-Hop antibody (D6E3, #5669, 1:1,000), anti-Hsp40 antibody (#4868, 1:3,000), anti-acetylated lysine (Lys-Ac) antibody (#9941, 1:1,000), anti-cytochrome *c* antibody (#11940, 1:3,000), anti-cox-4 antibody (#4844, 1:3,000), anti-caspase-9 antibody (#9508, 1:3,000), anti-cleaved caspase-3 antibody (#9661, 1:3,000) and anti-HDAC4 antibody (#7628, 1:3,000) were from Cell Signaling Technology. Anti-Hsp90 antibody (H-114, sc-7947, 1:3,000), anti-CHIP antibody (H-231, sc-66830, 1:3,000), anti-Myc antibody (9E10, sc-40, 1:3,000), anti-ARD1 antibody (FL-235, sc-33820, 1:3,000) and anti-GST antibody (B-14, sc-138, 1:3,000) were purchased from Santa Cruz. The anti-FLAG antibody (M2, F1804, 1:3,000) and anti-tubulin antibody (DM1A, T9016, 1:3,000) were from Sigma. Anti-GFP antibody (ab6556, 1:3,000) and anti-ubiquitin antibody (Z0458, 1:3,000) were from Abcam and Dako, respectively.

### Immunoblotting and immunoprecipitation

Proteins were extracted using lysis buffer consisting of 20 mM Tris (pH 7.5), 150 mM NaCl, 0.1 mM EDTA, 0.2% Triton X-100 and a protease inhibitor cocktail (Roche). Then, 20 μg of cell extracts were used for immunoblotting. For immunoprecipitation, 1 mg proteins were incubated with a corresponding primary antibody conjugated to A or G bead (Upstate) overnight at 4 °C. Beads were washed three times with washing buffer containing 20 mM Tris (pH 7.5), 150 mM NaCl and 0.1 mM EDTA. After one-dimensional SDS–PAGE, membranes were immunoblotted using the corresponding primary antibody overnight at 4 °C. HRP-conjugated secondary antibodies were incubated with the membranes for 1 h at room temperature. Visualization was performed using ECL Plus (Intron) and LAS-4000 (GE Healthcare). Uncroped blots are shown in Supplementary Fig. 12.

### Screening of binding proteins

The protocol of assay for protein-protein interaction was from previous study^49^. HEK293T cells were transiently transfected with FLAG-HA tagged ARD1. After two days, cells were lysed in lysis buffer consisting of 20 mM Tris (pH 7.5), 150 mM NaCl, 0.1 mM EDTA, 0.2% Triton X-100 and a protease inhibitor cocktail (Roche). Cell lysates were incubated with anti-M2 resin (Sigma) for 2 h at 4 °C. The resins were collected by centrifugation, and then washed three times with washing buffer, which consisted of 20 mM Tris (pH 7.5), 150 mM NaCl and 0.1 mM EDTA. Bound proteins were eluted by 3 × FLAG-peptide and immunoprecipitated again using anti-HA-antibody for 2 h at 4 °C. Bound proteins were analysed by SDS–PAGE and silver staining.

### *In vitro* acetylation assay

BL21 cells transformed with plasmids pGEX-4 T-1-ARD1 or pGEX-4 T-1-Hsp70 were grown to and OD600 of 0.6–0.8. Overall, 1 mM IPTG was added to induce GST-tagged Hsp70 or ARD1, then cells were grown for 4 h. Cells were collected and proteins were extracted with a lysis buffer containing 50 mM Tris–HCl (pH 8), 250 mM NaCl, 2.5 mM EDTA and 1% Triton X-100. GST-tagged proteins were purified with Glutathione Sepharose 4B (GE Healthcare) and followed by eluted with an elution buffer containing 50 mM Tris–HCl (pH 8) and 10 mM reduced glutathione (GSH). Acetylation assay was performed as described^29^. Briefly, 0.5–1 μg purified recombinant was incubated in the reaction mixture containing 50 mM Tris–HCl (pH 8), 0.1 mM EDTA, 1 mM DTT, 10% glycerol and 10 mM acetyl-CoA at 37 °C. Reaction products were separated by SDS–PAGE and analysed by western blotting using an anti-Lys-Ac antibody. Input proteins were visually quantified using Coomassie brilliant blue staining.

### *In vitro* deacetylation assay

After *in vitro* acetylation assay, GST-Hsp70 was pulled-down and washed three times with PBS. Hsp70 recombinant was pulled-downed and incubated with FLAG-HDAC4 immunoprecipitated from HEK293T cells in the reaction mixture containing 25 mM Tris–HCl (pH 8), 137 mM NaCl, 2.7 mM KCl and 1 mM MgCl_2_ at 30 °C for 1 h. Reaction products were separated by SDS–PAGE and analysed by western blotting. Input proteins were visually quantified using Coomassie brilliant blue staining.

### *In vitro*-binding assay

A total of 1 μg purified GST-Hsp70 and GST-ARD1 were subjected to *in vitro* acetylation assay for 1 h. GST-Hsp70 was pulled-down and washed with PBS three times. Pull-downed GST-Hsp70 was incubated with 1 μg human Hop (BioVision) and 0.5 μg human CHIP (BioVision) recombinants in binding buffer containing 50 mM Tris–HCl (pH 8), 0.1 mM EDTA, 1 mM DTT an 10% glycerol for 2 h at 4 °C. GST bead was pulled-down and washed with binding buffer three times. Hop and CHIP recombinants bound to GST-Hsp70 were eluted in SDS–PAGE sample buffer and analysed by western blotting.

### Mass spectrometric analysis

Mass spectrometric analysis was performed as previously described^28^. For enzymatic digestion of recombinant Hsp70, two GST-Hsp70 samples used in the *in vitro* acetylation assay (incubation with or without ARD1) were immediately denatured using 6 M GuHCl and reduced with 5 mM DTT for 30 min at room temperature, followed by alkylation with 25 mM iodoacetamide for 30 min. Glu-C digestions were carried out for 6 h at 25 °C and the reactions were quenched by 5% formic acid.

For micro RPLC–MS/MS analysis, Glu-C digested protein samples were loaded onto fused silica capillary columns (100 μm i.d., 360 μm o.d.) containing 8 cm of 5 μm particle size Polaris C-18 column material (Metachem). The column was placed in-line with an Agilent HP1100 quaternary LC pump and a splitter system was used to achieve a flow rate of 250 nl min^−1^. Buffer A (5% acetonitrile and 0.1% formic acid) and buffer B (80% acetonitrile and 0.1% formic acid) were used to make a 80 min gradient. The gradient profile started with 5 min of 100% buffer A, followed by a 60 min gradient from 0 to 55% buffer B, a 10 min gradient from 55 to 100% buffer B and a 10 min gradient of 100% buffer B. Eluted peptides were directly electrosprayed into an LTQ Ion Trap Mass spectrometer (ThermoFinnigan) by applying 2.3 kV of DC voltage. A data-dependent scan consisting of one full MS scan (400–1,400 *m*/*z*) and 10 data-dependent MS/MS scans were used to generate the MS/MS spectra of the eluted peptides. A normalized collision energy of 35% was used throughout the data acquisition.

### ATP-binding assay

A total of 0.1 μg recombinant proteins subjected into *in vitro* acetylation assay or 1 mg cell extracts were incubated with ATP-agarose beads (Innova Biosciences) in a binding buffer containing 20 mM Tris–HCl (pH 7.4), 150 mM NaCl, 1 mM EDTA and 10 mM MgCl_2_ for 2 h at 4 °C. Then, the ATP-agarose was washed three times with the same buffer. Proteins bound to ATP-agarose were eluted in SDS–PAGE sample buffer and analysed by western blotting.

### ATPase activity assay

ATP hydrolysis was measured using an ATPase Assay Kit (Innova Biosciences) following the manufacturer's instructions. 1 μg Hsp70 and Hsc70 recombinant proteins used in the *in vitro* acetylation assay with or without 1 μg ARD1 recombinant were incubated with 1 μg Hsp40 recombinant (ATGen) in a reaction buffer consisting of 50 mM Tris (pH 7.5), 2.5 mM MgCl_2_ and 0.5 mM ATP at room temperature. For cells, GFP-Hsp70 precipitated from 1 mg cell extracts was used for reaction. After 60 min, PiColorLock Gold reagent and Accelerator were added to the solution. Stabilizer was added 2 min later, and the resulting green colour was allowed to develop for 30 min at room temperature. Absorbance was measured at 595 nm.

### Cell fractionation

Cytosolic/mitochondrial fractionation were prepared using Mitochondiral Isolation Kit for Cultured Cells (ThermoFisher Scientific) according to manufacturer's instruction. The resulting fractionated proteins were used for western blotting.

### Luciferase refolding assay

Luciferase refolding assay was performed as previously described with some modifications (Supplementary Fig. 7)^47^,^50^. Cells were transfected with equal amounts of pRSV-luciferase. After 24 h, protein synthesis was inhibited by treating the cells with cycloheximide (Sigma) for 30 min. Thereafter, protein unfolding was induced by 30 min H_2_O_2_ treatment. After 2 h of recovery, the firefly luciferase activity was measured, and the relative value to the initial (0 h) activity was calculated. Luciferase activity was normalized to the total protein concentration.

### Protein aggregation assay

Cells expressing wild-type or mutant Hsp70 were exposed to 1 mM of H_2_O_2_ for 24 h and protein aggregation was measured using the Proteostat Protein Aggregation Assay Kit (Enzo Lifescience) following the manufacturer's instructions.

### Quantification of protein degradation

Ubiquitin-mediated protein degradation was assessed by quantification of protein udiquitination as described previously^51^. Cells expressing wild-type or mutant Hsp70 were treated with 1 mM H_2_O_2_ for 20 h. Before lyse the cell, MG132, a proteasome inhibitor, was treated for 3 h and protein extracts were subjected into western blot analysis for the detection of ubiqutinated proteins.

### Protein sequence alignment and structural analysis

Hsp70 protein sequence alignment was conducted by Clustal Omega programme. The structures of ADP- and ATP-bound *E.coli* DnaK were collected from the Protein Data Bank (PDB) and the position of the R76 residue in ADP- and ATP-bound DnaK (PDB ID: 2KHO and 4JNE, respectively) was mapped using PyMOL software.

### Cell viability and apoptosis assay

Cell viability was calculated by measuring the amount of lactate dehydrogenase (LDH) released from the cells into the medium. Conditioned media from cultured cells were collected, and LDH activities were determined with an LDH assay kit (DoGen). Total cellular LDH activity was measured by solubilizing the cells with 0.2% Triton X-100.

To quantify apoptotic cells, fluorescence-activated cell sorting (FACS) was used after Annexin V-allophycocyanin (APC) and 7-amino-actinomycin D (7-AAD) labelling as described in the manufacturer's protocol (APC Annexin V Apoptosis Detection Kit, Biolegends). All FACS analyses for apoptosis detection were performed in triplicates.

### *In vitro* mRNA transcription

For the co-expression of Hsp70 and ARD1 in zebrafish, Hsp70 and ARD1 were transcribed from linearized expression constructs (pEGFP-C3-Hsp70 and pCS2^+^-ARD1) using the mMESSAGe mMACHINE Kit (Ambion) following the manufacturer's instructions. Zebrafish embryos were injected with 200 pg of total mRNA.

### Immunohistochemistry

To induce dopaminergic neuron damage, zebrafish was treated with 1-methyl-4-phenyl-1,2,3,6-tetrahydropyridine (MPTP) (10 μg ml^−1^) at 1–4 dpf 52,53. For whole-mount immunohistochemistry, embryos were fixed in 4% paraformaldehyde overnight at 4 °C and then transferred to cold methanol. After overnight incubation at −20 °C, embryos were permeabilized with acetone at −20 °C for 5 min, rehydrated and washed with PBS containing 0.1% Tween 20. Embryos were then blocked with 5% bovine serum albumin and 10% normal goat serum in PBDTT consisting of 1 × PBS, 1% dimethyl sulfoxide, 0.5% Triton X-100 and 0.1% Tween 20, and incubated overnight at 4 °C with anti-TH antibody (Millipore). After the embryos were washed with PBDTT, they were incubated with biotin-labeled secondary antibody (Vector Labs) and detected with VECTASTAIN ABC Kit (Vector Labs) following the manufacturer's instructions. The specimens were imaged using a Leica DM5000B microscope with Leica Application Suite V4.2 software (Leica).

### Zebrafish survival rate

Zebrafish survival assay was carried out as described previously with some modifications^54^,^55^. 3dpf zebrafish embryos were placed into petri dishes and H_2_O_2_ was added to a final concentration of 5 mM. After 1 h exposure, embryos were washed with fresh embryo buffer and incubated at 28.5 °C for 24 h before counting for survival.

### Behavioural analysis

Behavioural analysis was performed as described previously with some modifications^53^,^56^,^57^ Zebrafish was treated with MPTP (10 μg ml^−1^) at 1–5 dpf and the locomotor activity was observed at 5 dpf. The light intensity of the observation chamber was set to 4,400 lux. Zebrafish were individually placed into each well of a 24 well plate, and allowed to accommodate for 5 min before recording. Spontaneous free-swimming was recorded for 10 min period using the DanioVision tracking system (Noldus Information Technology), which included a high-resolution digital video camera. Locomotor activity was analysed by calculating the total distance travelled using EthoVision XT 8 Locomotion Tracking software (Noldus Information Technology). The behavioural analysis was carried out between 9:00 and 11:00 AM.

### Statistical analysis

Results are expressed as the mean's±s.d's or±s.e.m's. *P* values were calculated by applying the two-tailed Student's *t* test or one-way ANOVA. A difference was considered statistically significant at a value of *P*<0.05.

### Data availability

The authors declare that all data supporting the findings of this study are available within the article and its Supplementary Information files. Any additional relevant information can be obtained from the authors on request.

## Additional information

**How to cite this article:** Seo, J. H. *et al*. ARD1-mediated Hsp70 acetylation balances stress-induced protein refolding and degradation. *Nat. Commun.* **7,** 12882 doi: 10.1038/ncomms12882 (2016).

## Supplementary Material

Supplementary Information Supplementary Figures 1 - 12

Peer Review File
