# Supplementary material for: ARD1-mediated Hsp70 acetylation balances stress-induced protein refolding and degradation
Source: Nat Commun. 2016 Oct 6;7:12882. doi: 10.1038/ncomms12882 (PMC5059642; doi:10.1038/ncomms12882)
Supplement: Supplementary Information — Supplementary Figures 1 - 12 [file ncomms12882-s1.pdf]

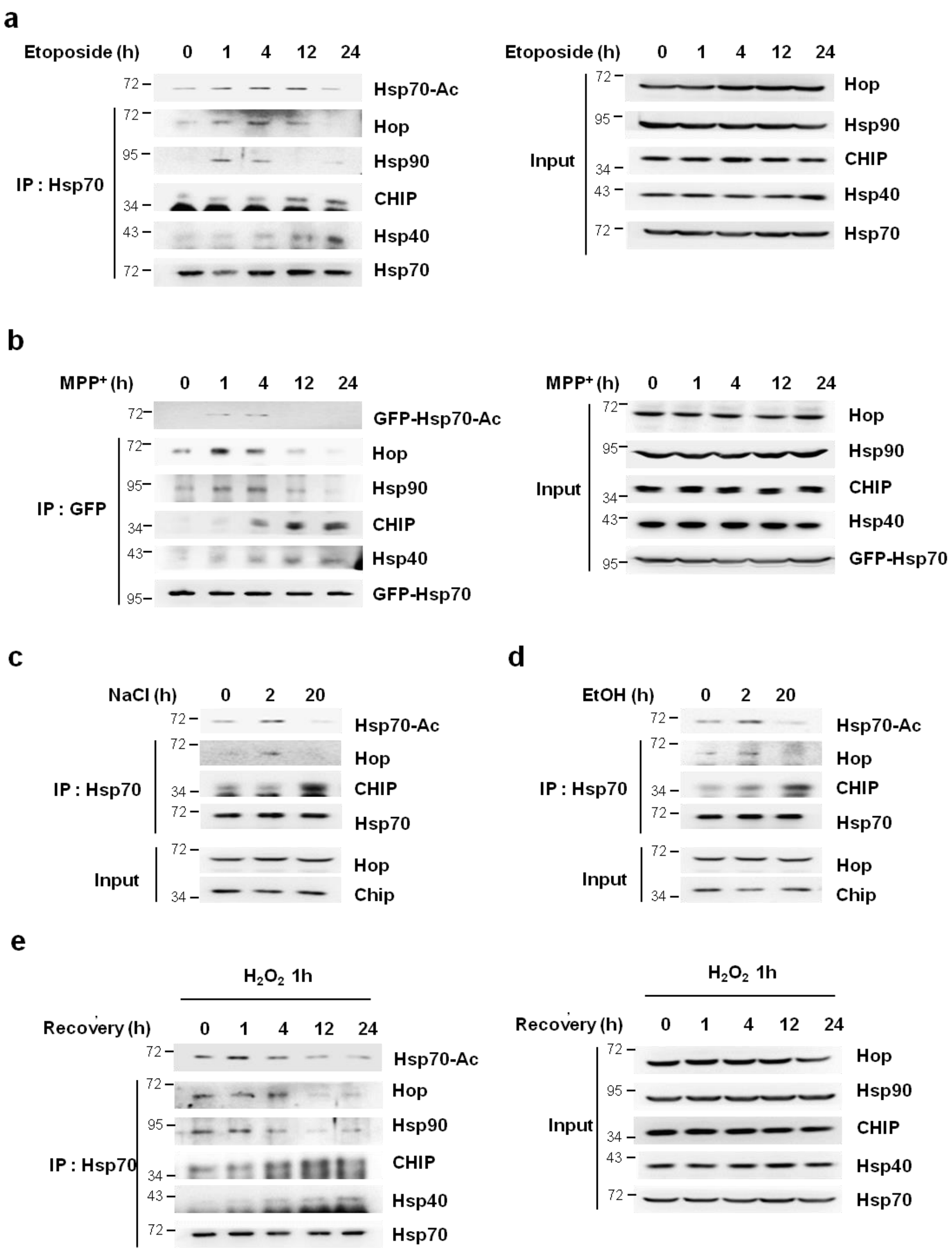

Supplementary Figure 1. The Hsp70 acetylation level is related to the co-chaperone binding of Hsp70 under various stress conditions.

(a) Etoposide treatment gradually changes acetylation level and co-chaperone complexes of Hsp70. After treating HEK293T cells with 100 mM etoposide, endogenous Hsp70 was precipitated, and its co-chaperone binding partners were analyzed by western blotting. Acetylation of endogenous Hsp70 was accessed by immunoprecipitation using an anti-Lys-Ac antibody.

(b) MPP^+ treatment gradually changes acetylation level and co-chaperone bindings of Hsp70. After SH-SY5Y cells stably expressing GFP-Hsp70 were treated with 1 mM MPP^+, GFP-Hsp70 was precipitated using an anti-GFP antibody, and its co-chaperone complexes were determined by western blotting. Acetylation of GFP-Hsp70 was analyzed by immunoprecipitation using an anti-Lys-Ac antibody.

(c) NaCl treatment changes acetylation level and co-chaperone complexes of Hsp70. HEK293T cells were treated with 100 mM NaCl for the indicated time. Acetylation of endogenous Hsp70 was accessed by immunoprecipitation using an anti-Lys-Ac antibody. For co-chaperone binding, endogenous Hsp70 was immunoprecipitated using an anti-Hsp70 antibody and its binding to Hop and CHIP was analyzed by western blotting.

(d) EtOH treatment changes acetylation level and co-chaperone complexes of Hsp70. After treating HEK293T cells with 150 mM EtOH, endogenous Hsp70 was precipitated, and its co-chaperone binding partners were analyzed by western blotting. Acetylation of endogenous Hsp70 was accessed by immunoprecipitation using an anti-Lys-Ac antibody.

(e) After brief expose of cellular stress, Hsp70 gradually alters its acetylation level and co-chaperone binding partners. HEK293T cells were treated with 1 mM H_2O_2 for 1 h. During the recovery time, the temporal changes in acetylation and co-chaperone binding of endogenous Hsp70 were assessed by immunoprecipitation and western blotting.

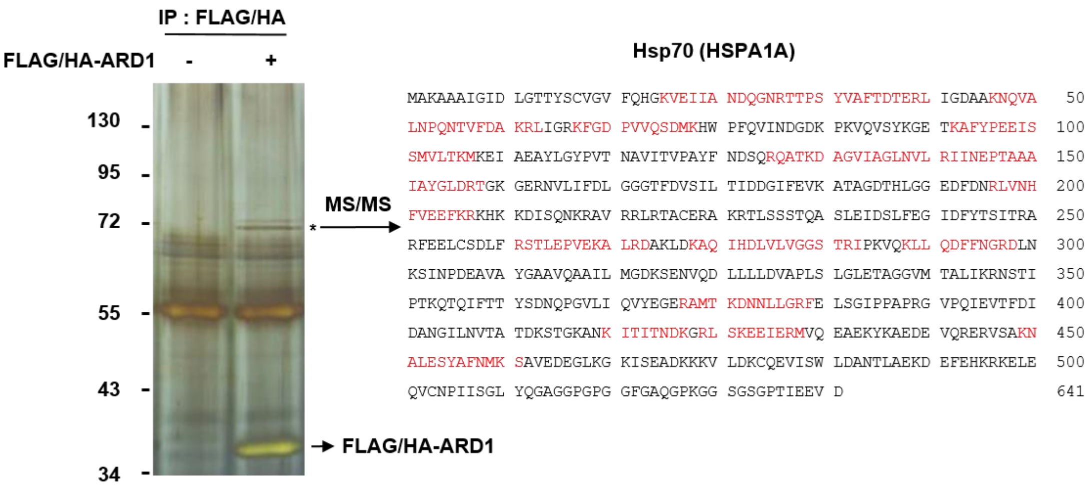

Supplementary Figure 2. ARD1 binds to Hsp70.

Hsp70 binding to FLAG-HA-ARD1 was identified by mass spectrometry. The Hsp70 band is indicated by an asterisk (*), and Hsp70 peptide sequences identified by mass spectrometry are indicated in red.

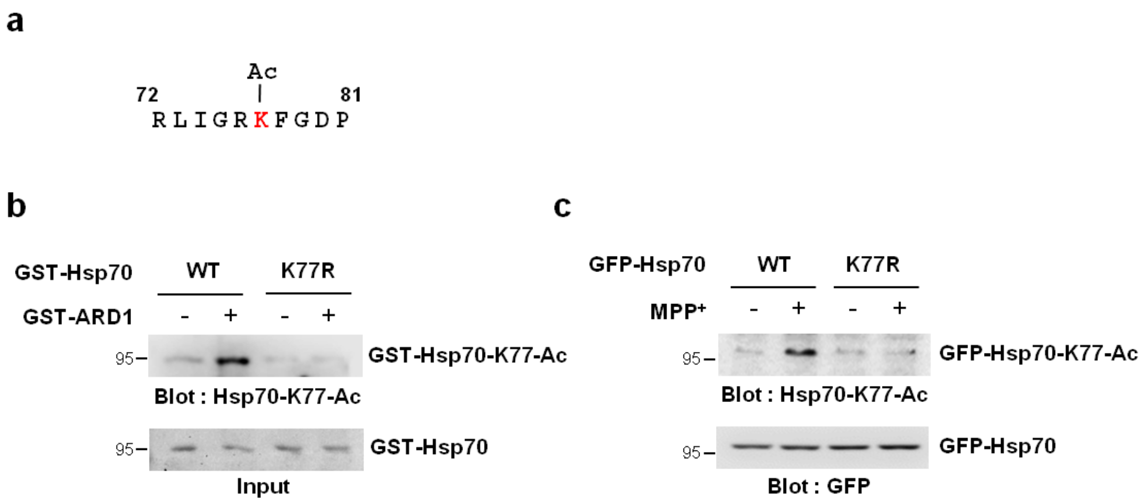

Supplementary Figure 3. Construction of an antibody specific for K77 acetylation of Hsp70.

(a) Antigen sequence for anti-Hsp70-K77-Ac antibody.

(b) The constructed anti-Hsp70-K77-Ac antibody recognizes the K77 acetylation of Hsp70 *in vitro*. GST-ARD1, GST-Hsp70 WT and GST-Hsp70 K77R recombinants were subjected to *in vitro* acetylation assays. Acetylation levels of GST-Hsp70 were analyzed by western blotting.

(c) The stress-induced increase in Hsp70 acetylation is detected using anti-Hsp70-K77-Ac antibody in cells. SH-SY5Y cells stably expressing GFP-Hsp70 WT and GFP-Hsp70 K77R were treated with MPP^+ for 1 h. Acetylation levels of GFP-Hsp70 were assessed by western blotting.

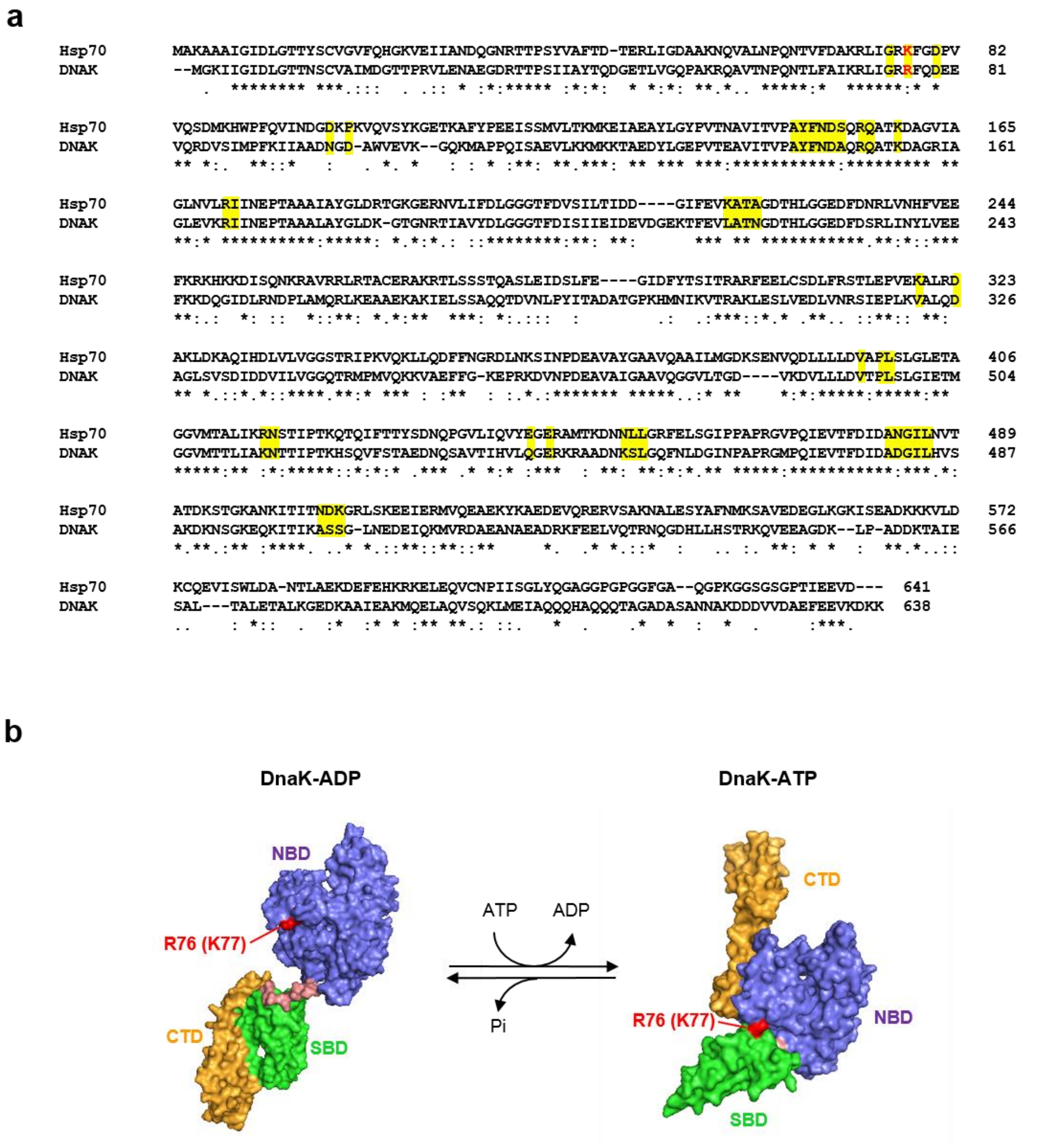

Supplementary Figure 4. Hsp70 acetylation occurs at the NBD-SBD interface.

(a) K77 of Hsp70 corresponds to R76 of DnaK. The contacting residues at the NBD-SBD interface are highlighted in yellow. The asterisk (*) at the bottom line of the alignment indicates identical residues in a given sequence position, whereas double (:) and single (.) dots refer to strongly and weakly similar residues, respectively.

(b) Acetylation occurs at the NBD-SBD interface of ATP-bound Hsp70. During the ATPase cycle, R76 in the NBD interacts with the SBD when DnaK is bound to ATP. R76 is marked in red in ADP- (PDB: 2KHO) and ATP-bound (PDB: 4JNE) DnaK.

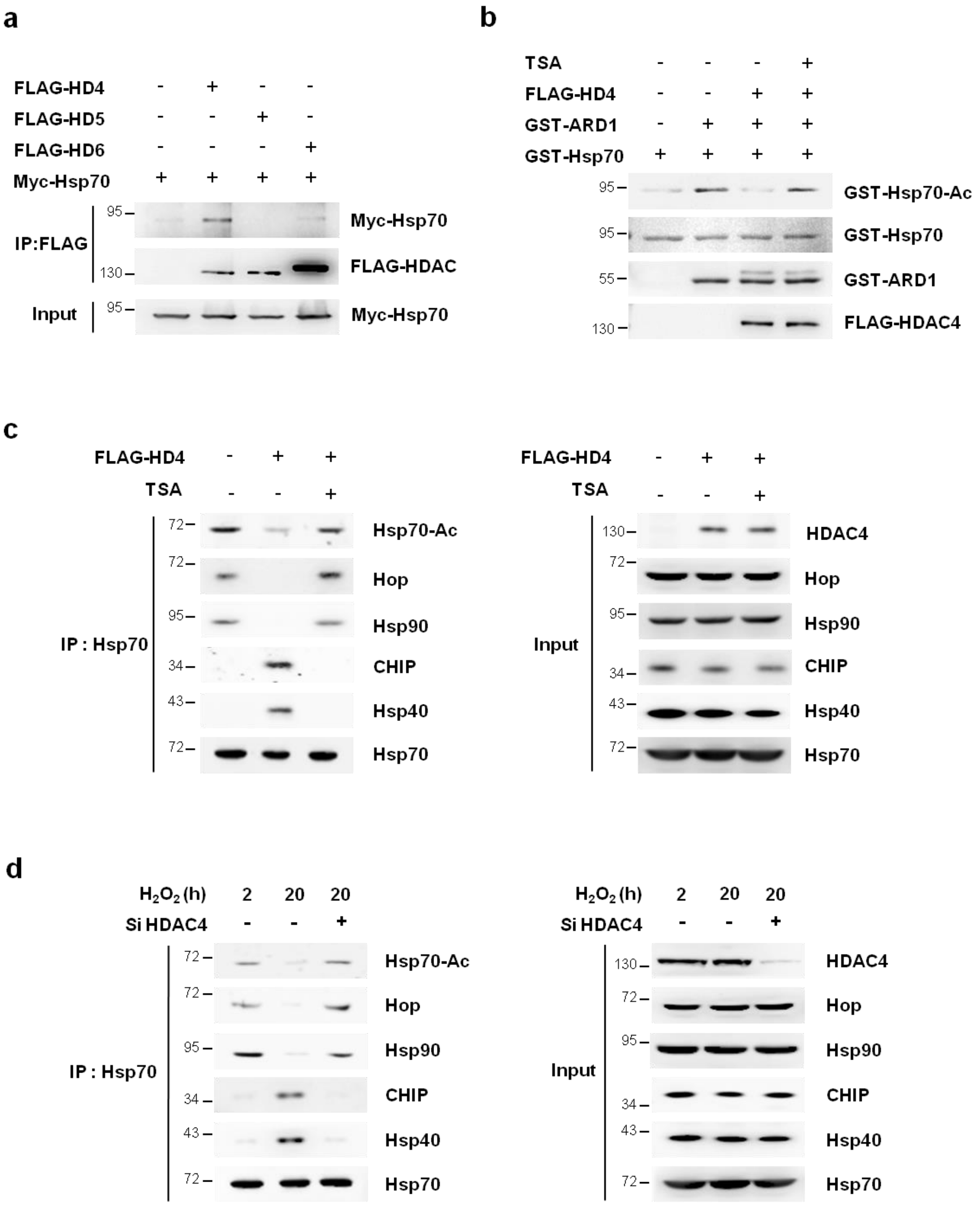

Supplementary Figure 5. Hsp70 is deacetylated by HDAC4.

(a) HDAC4 binds to Hsp70. After FLAG-HDACs and Myc-Hsp70 were transfected into HEK293T cells, FLAG-HDACs were precipitated from HEK293T cells, and co-precipitation

of Myc-Hsp70 was assessed.

(b) HDAC4 deacetylates HSP70 *in vitro*. After GST-Hsp70 was acetylated by GST-ARD1 *in vitro*, FLAG-HDAC4 precipitated from HEK293T cells was subjected to *in vitro* deacetylation assays. Acetylation levels of GST-Hsp70 were analyzed by western blotting using an anti-Lys-Ac antibody.

(c) HDAC4 changes the co-chaperone binding pattern of Hsp70. HEK293T cells expressing HDAC4 were treated with 1 mM H_2O_2 for 1 h with or without TSA treatment for 4 h. Endogenous Hsp70 was precipitated, and its acetylation and co-chaperone bindings were assessed by western blotting.

(d) HDAC4 is a relevant deacetylase to K77 acetylation of Hsp70. HEK293T cells transfected with HDAC4 siRNA were treated with 1 mM H_2O_2 for the indicated times. Endogenous Hsp70 was precipitated, and its acetylation and co-chaperone binding was assessed by western blotting.

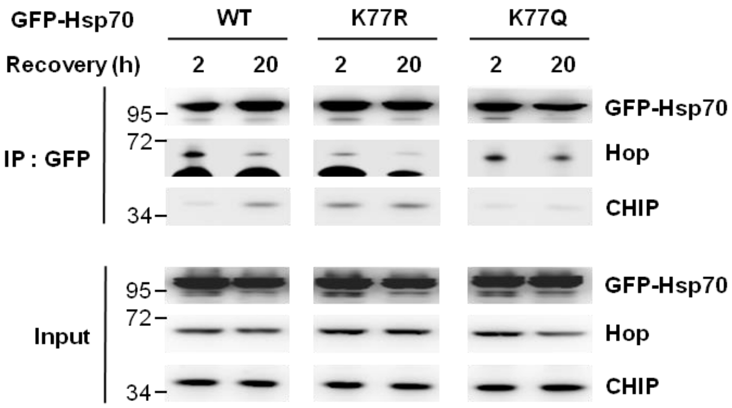

Supplementary Figure 6. During the recovery time after stress, Hsp70 switches its co-chaperone binding depending on the acetylation/deacetylation state of K77 residue.

HEK293T cells expressing GFP-Hsp70 WT, K77R and K77Q were treated with 1 mM H_2O_2 for 1 h, followed by washing with phosphate-buffered saline. After 2 h and 20 h, co-chaperone binding of GFP-Hsp70 was determined by immunoprecipitation using an anti-GFP antibody.

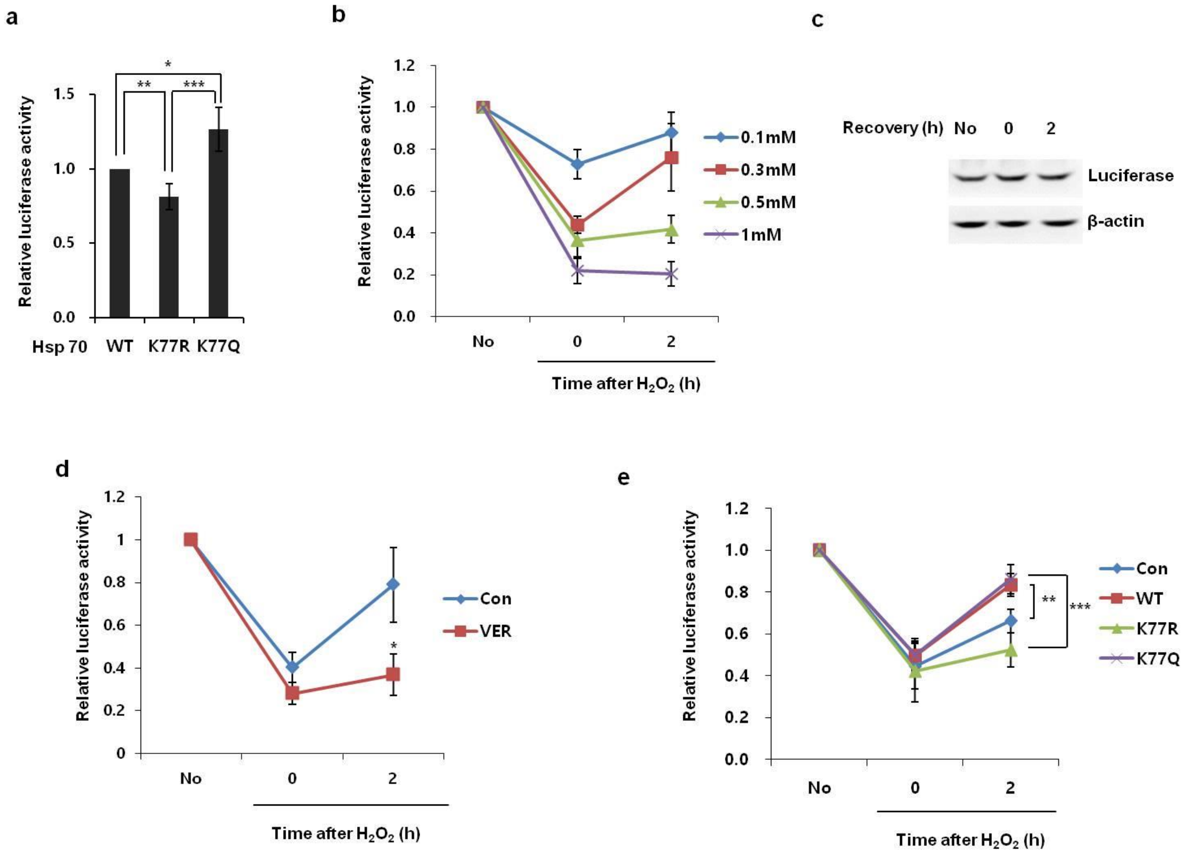

Supplementary Figure 7. Hsp70 acetylation is required for protein refolding in the early phase after stress induced by heat shock or hydrogen peroxide.

(a) Acetylated Hsp70 encourages protein refolding in the early phase after heat shock stress. HEK293T cells were transfected with the indicated Hsp70 plasmids. After treatment at 42 °C heat shock for 30 min, the cells were recovered for 4 h, and then protein refolding was measured.

(b-d) Establishment of the luciferase refolding assay using H_2O_2-induced protein denaturation. (b) Luciferase protein is refolded after 0.3 mM H_2O_2-induced stress. After the HEK293T cells were treated with the indicated concentration of H_2O_2 for 30 min, the cells were recovered for 2 h and then protein refolding was measured. (c) Total amount of soluble luciferase is not changed during protein refolding after 0.3 mM H_2O_2-induced stress. HEK293T cells were treated with 0.3 mM H_2O_2 for 30 min and then the cells were recovered for 2 h. Cell lysates were extracted and the total amount of soluble luciferase was analyzed by

western blotting. (d) Hsp70 is required for protein refolding in the early phase after H_2O_2-induced stress. HEK293T cells were pretreated with Hsp70 inhibitor, VER-155008, for 30 min before H_2O_2 stimulation. After treatment with 0.3 mM H_2O_2 for 30 min with or without VER-155008, the cells were recovered for 2 h and protein refolding was measured.

(e) K77 acetylation of Hsp70 is necessary for protein refolding in the early phase after H_2O_2-induced stress. HEK293T cells were transfected with the indicated Hsp70 plasmids. After treatment with 0.3 mM H_2O_2 for 30 min, the cells were recovered for 2 h and protein refolding was measured.

Error bars indicate S.D. (n = 3). *$P < 0.05$; **$P < 0.01$; ***$P < 0.005$, *t* test. No; untreated, VER; VER-155008.

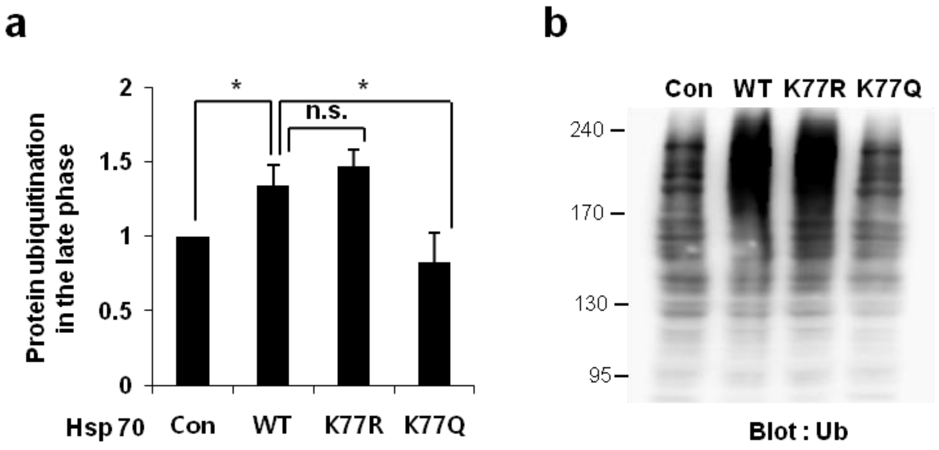

Supplementary Figure 8. Deacetylated Hsp70 controls protein ubiquitination in the late phase after brief stress.

K77Q mutation of Hsp70 impaired protein degradation in the late recovery phase after brief stress (20 h). HEK293T cells were treated with 1 mM H_2O_2 for 1 h, and then washed with phosphate-buffered saline. After 20 h, total protein ubiquitination was analyzed to estimate the amount of protein degradation. Protein ubiquitination was measured by western blotting using an anti-ubiquitin antibody (b) and quantified (a).

Error bars indicate S.D. (n = 3). *P < 0.05, *t* test. n.s.; not significant.

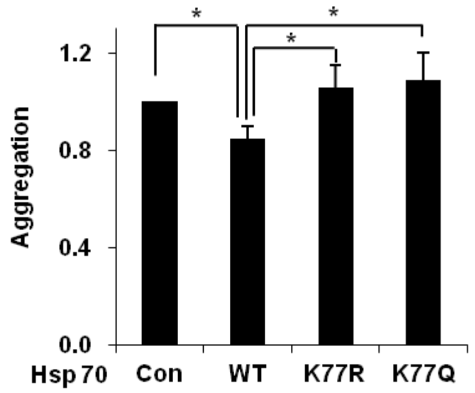

Supplementary Figure 9. The switch between Hsp70 acetylation/deacetylation is required to maintain protein homeostasis after brief stress.

Stress-induced protein aggregation was enhanced by both the K77R and K77Q mutations in Hsp70. HEK293T cells were treated with 1 mM H_2O_2 for 1 h, and then washed with phosphate-buffered saline to remove H_2O_2. After 24 h, the accumulation of protein aggregation was analyzed using protein aggregation detection dye.

Error bars indicate S.D. (n = 3). *$P < 0.05$, *t* test. n.s.; not significant.

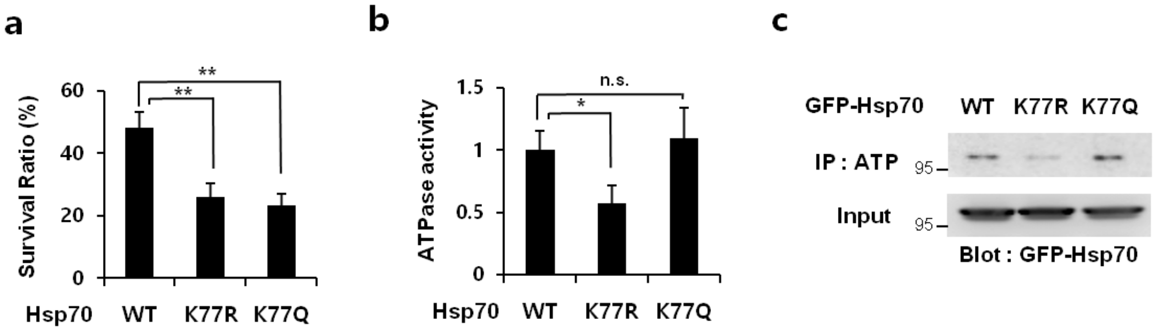

Supplementary Figure 10. K77R and K77Q mutations of Hsp70 reduce cell survival in response to stress regardless of their ATPase cycles.

(a) K77R and K77Q mutations of Hsp70 decrease the cell survival under stress condition. SH-SY5Y cells expressing GFP-Hsp70 WT, K77R and K77Q were stimulated with 1 mM H_2O_2 for 24 h, and the survival ratios were measured.

(b and c) . Unlike the K77R mutation, the K77Q mutation has no effect on the ATPase cycles of Hsp70. GFP-Hsp70 WT, K77R, and K77Q were transfected into HEK293T cells. GFP-Hsp70 WT, K77R, and K77Q were precipitated and the ATPase activities were measured (b). ATP binding affinities of GFP-Hsp70 WT, K77R, and K77Q were analyzed by immunoprecipitation using ATP-agarose beads (c).

Error bars indicate S.D. (n=3). *P < 0.05; **P < 0.01, *t* test. n.s.; not significant.

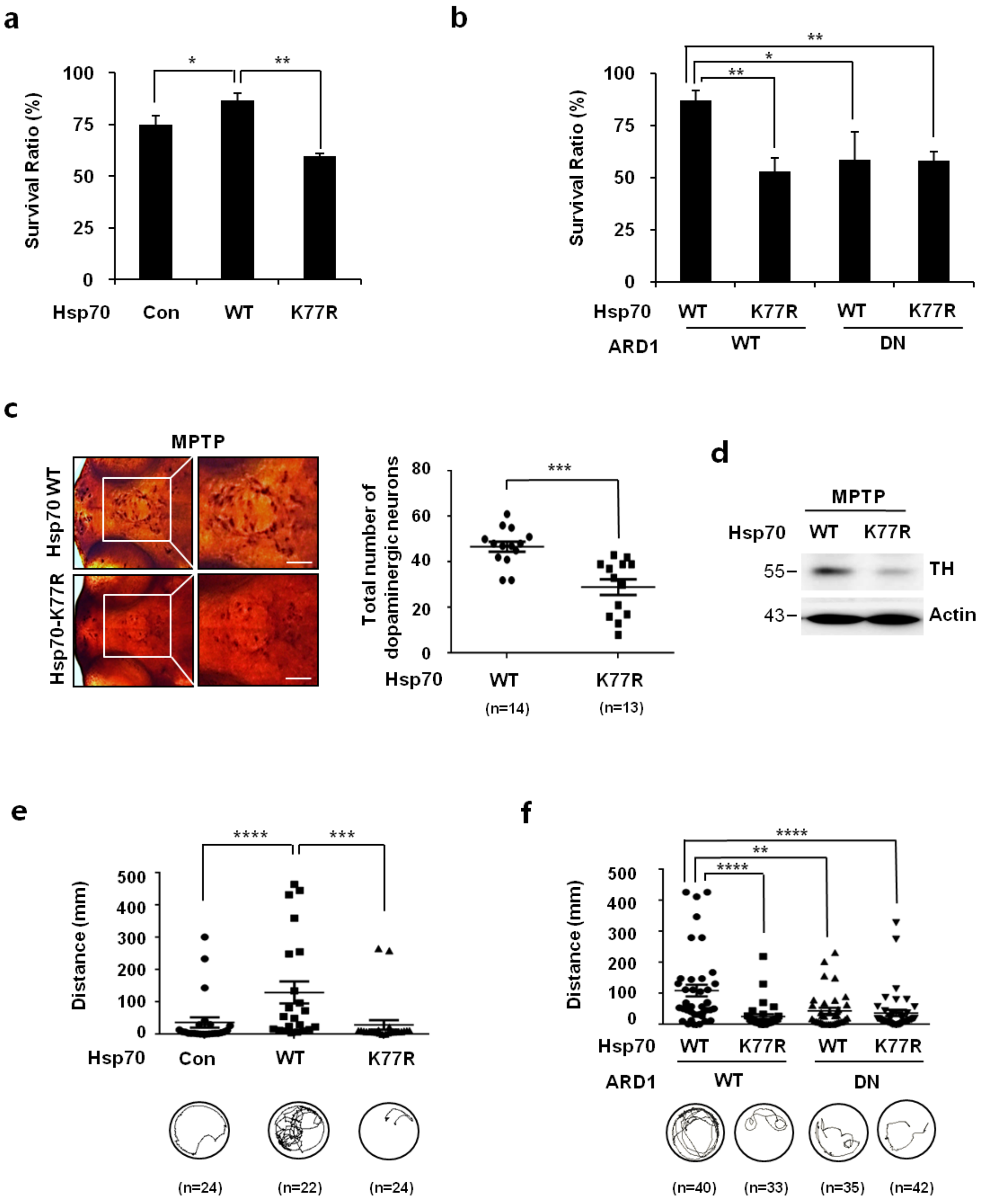

Supplementary Figure 11. ARD1-mediated Hsp70 acetylation protects cells against stress conditions *in vivo*.

(a) Hsp70 acetylation rescues zebrafish lethality. Zebrafish expressing GFP-Hsp70 WT and K77R were incubated with 5 mM H_2O_2 for 1 h at 3 dpf, and the survival ratios of zebrafish

were measured on the following day.

(b) ARD1 increases zebrafish survival through Hsp70 acetylation. WT or DN Myc-ARD1 were co-expressed with GFP-Hsp70 in zebrafish. At 3 dpf, embryos were treated with 5 mM H_2O_2 for 1 h, and the survival ratios were measured on the following day.

(c and d) Hsp70 acetylation protects doparminergic neurons against MPTP-induced cell death. Zebrafish expressing GFP-Hsp70 WT and K77R were treated with 10 ug/ml MPTP at 1–4 dpf. Total number (c) and expression level (d) of zebrafish dopaminergic neurons were measured by the dopaminergic neuron marker tyrosine hydroxylase (TH). TH-positive neuron in the diencephalon region of zebrafish embryos were immunostained (left) and counted (right) (c). Scale bars, 50 μm. TH expression in embryo were analyzed by western blotting (d).

(e) Hsp70 acetylation enhances the locomotor activity of zebrafish. Zerafish expressing GFP-Hsp70 WT and K77R were treated with MPTP 10 ug/ml at 1–5 dpf, then the locomotor activities of zebrafish were measured at 5 dpf (top). Embryos were adjusted to chamber for 5 min and tracked for 10 min. Representative swimming patterns of MPTP-treated zebrafish (bottom).

(f) ARD1 improves the locomotor activity of zebrafish through Hsp70 acetylation. Zebrafish co-expressing Myc-ARD1 and GFP-Hsp70 were treated with 10 ug/ml MPTP at 1–5 dpf, the locomotor activities of zebrafish were measured at 5 dpf (top). Embryos were adjusted to chamber for 5 min and tracked for 10 min. Representative swimming patterns of MPTP-treated zebrafish (bottom).

Error bars indicate S.D. (n = 3) (a and b) and S.E.M. The number (n) of the embryos examined for quantitative analyses is indicated at the bottom (e and f). $^*P < 0.05$; $^{**}P < 0.005$; $^{***}P < 0.001$, $^{****}P < 0.0001$, *t* test (a–c), one-way ANOVA (e and f).

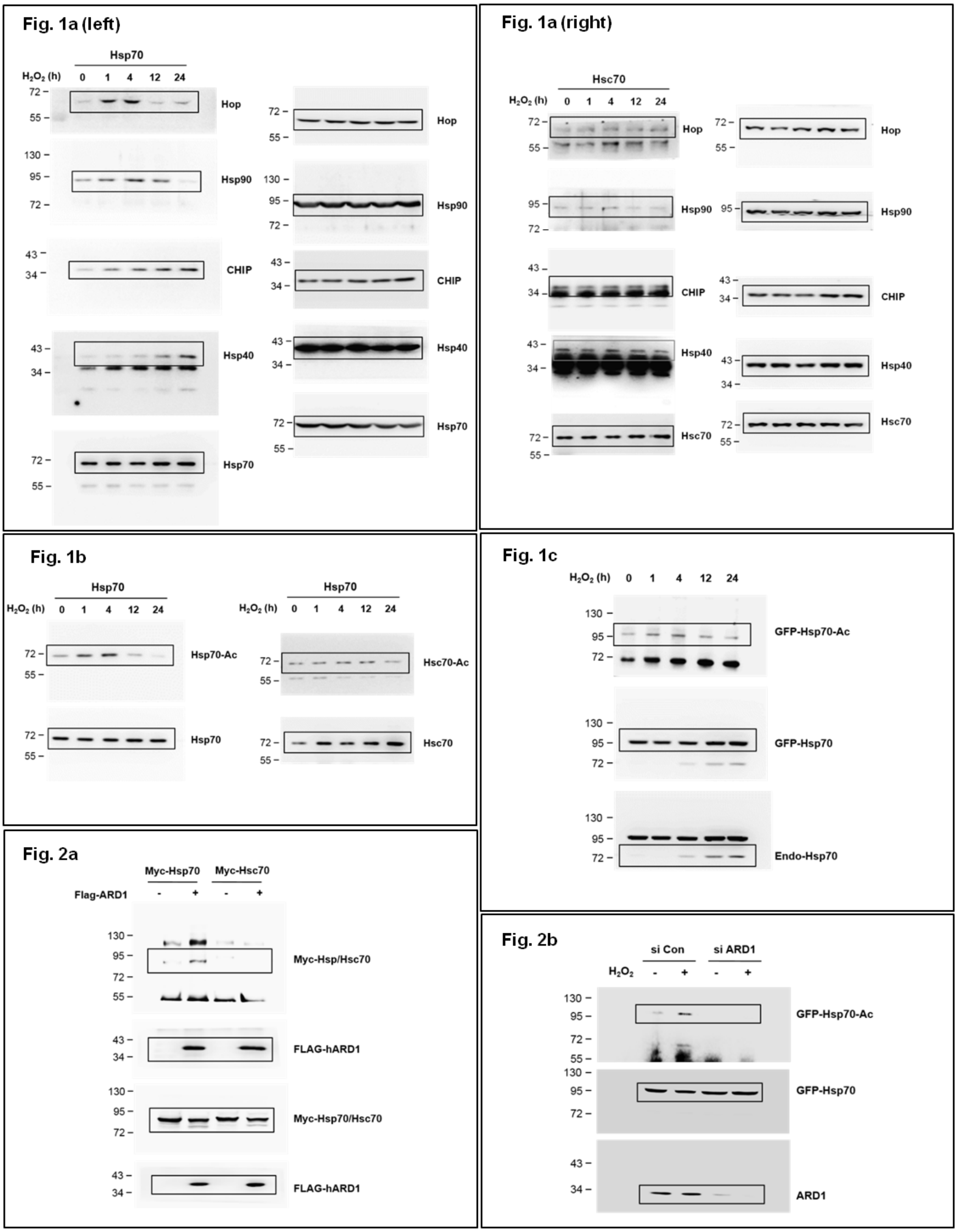

Supplementary Figure 12. Original images of western blots. Black boxes show approximate image used for presentation.

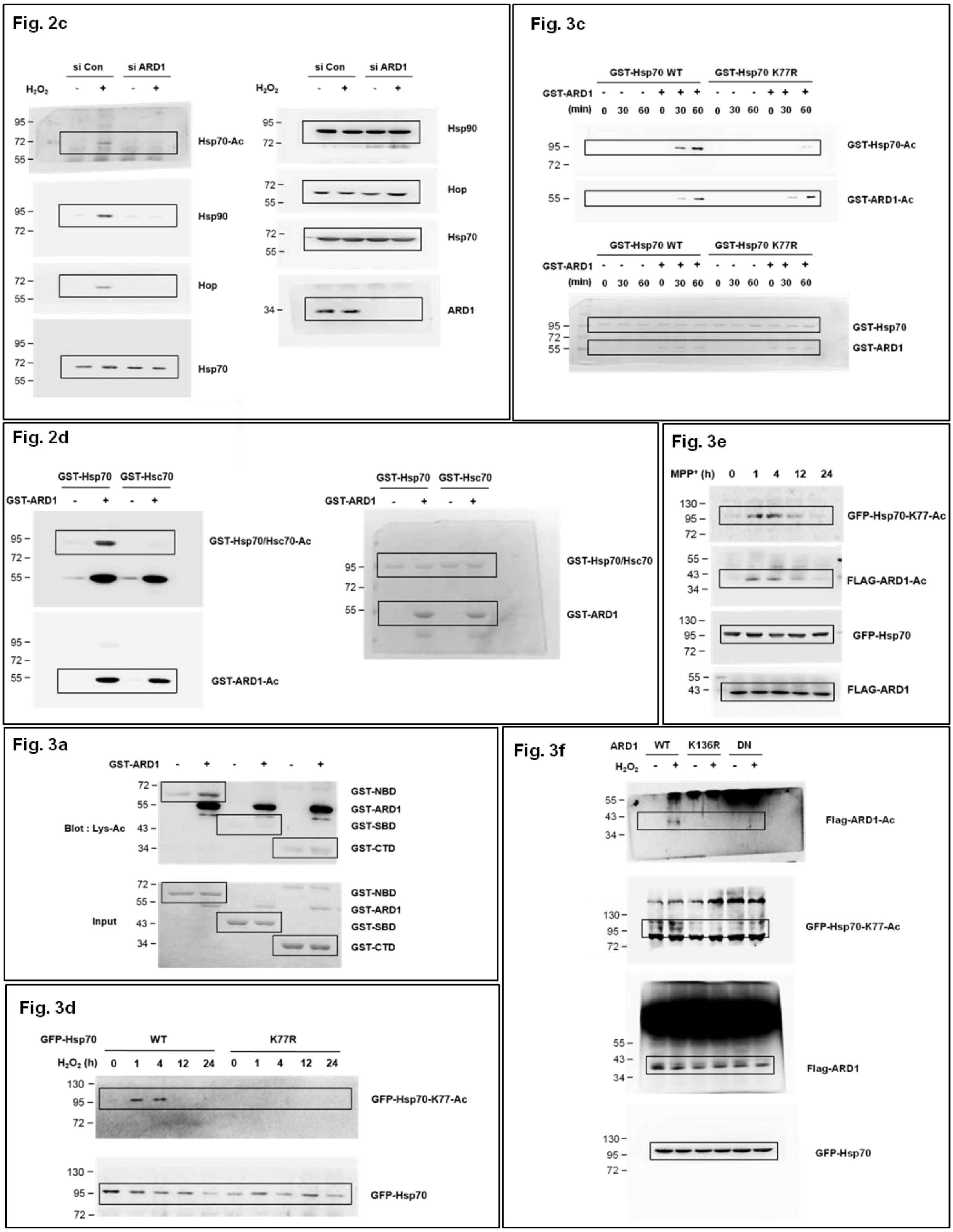

Supplementary Figure 12. Continued

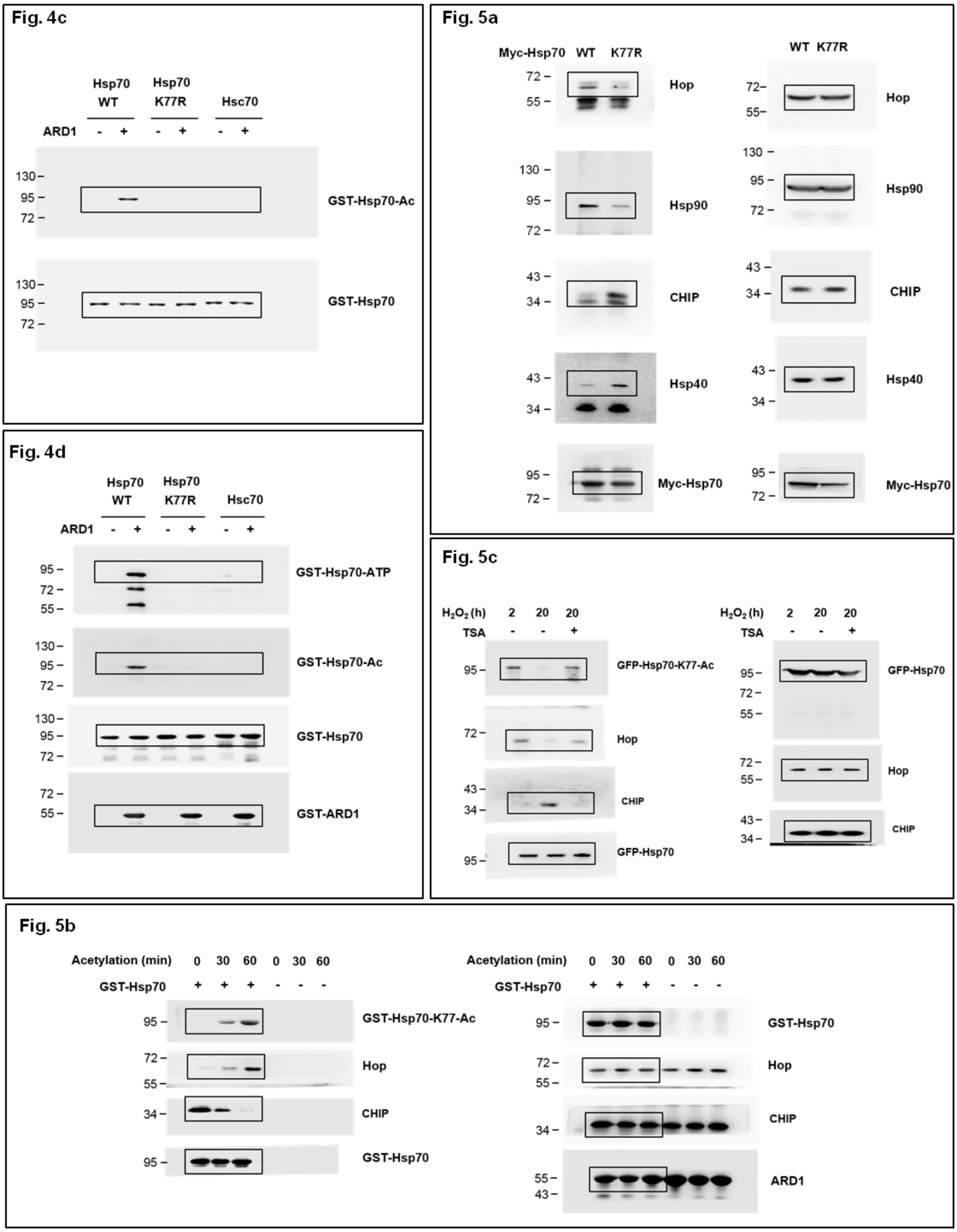

Supplementary Figure 12. Continued

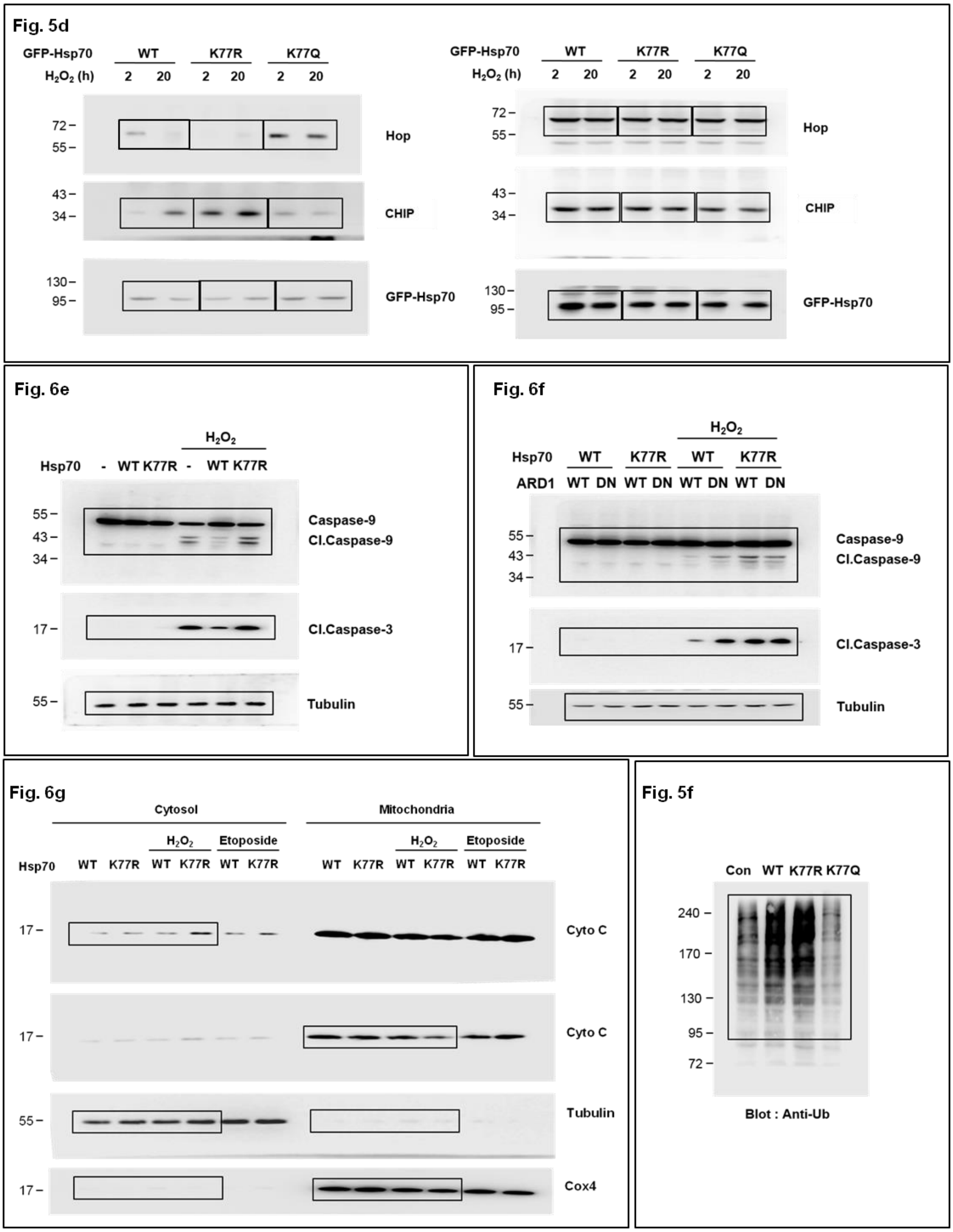

Supplementary Figure 12. Continued

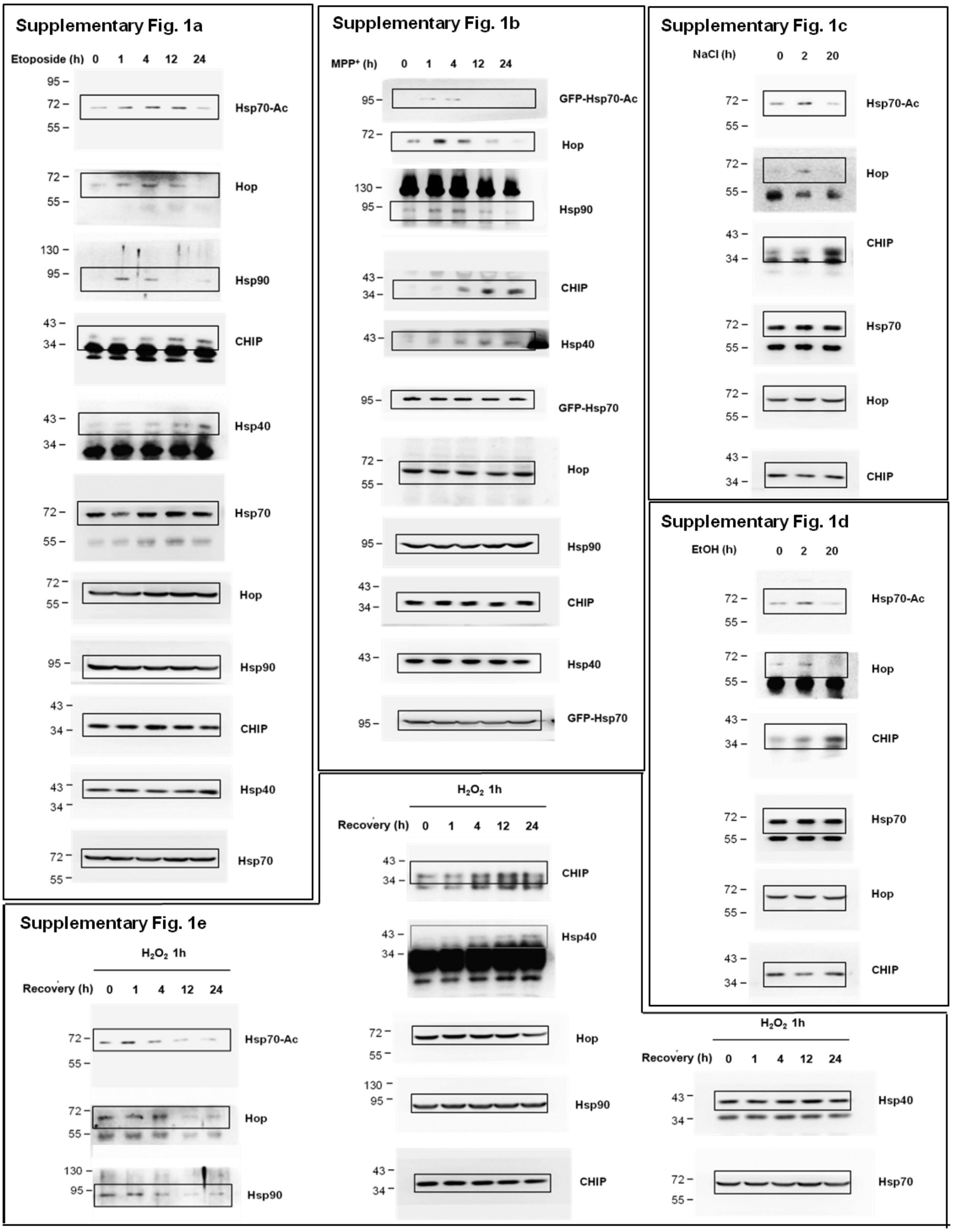

Supplementary Figure 12. Continued

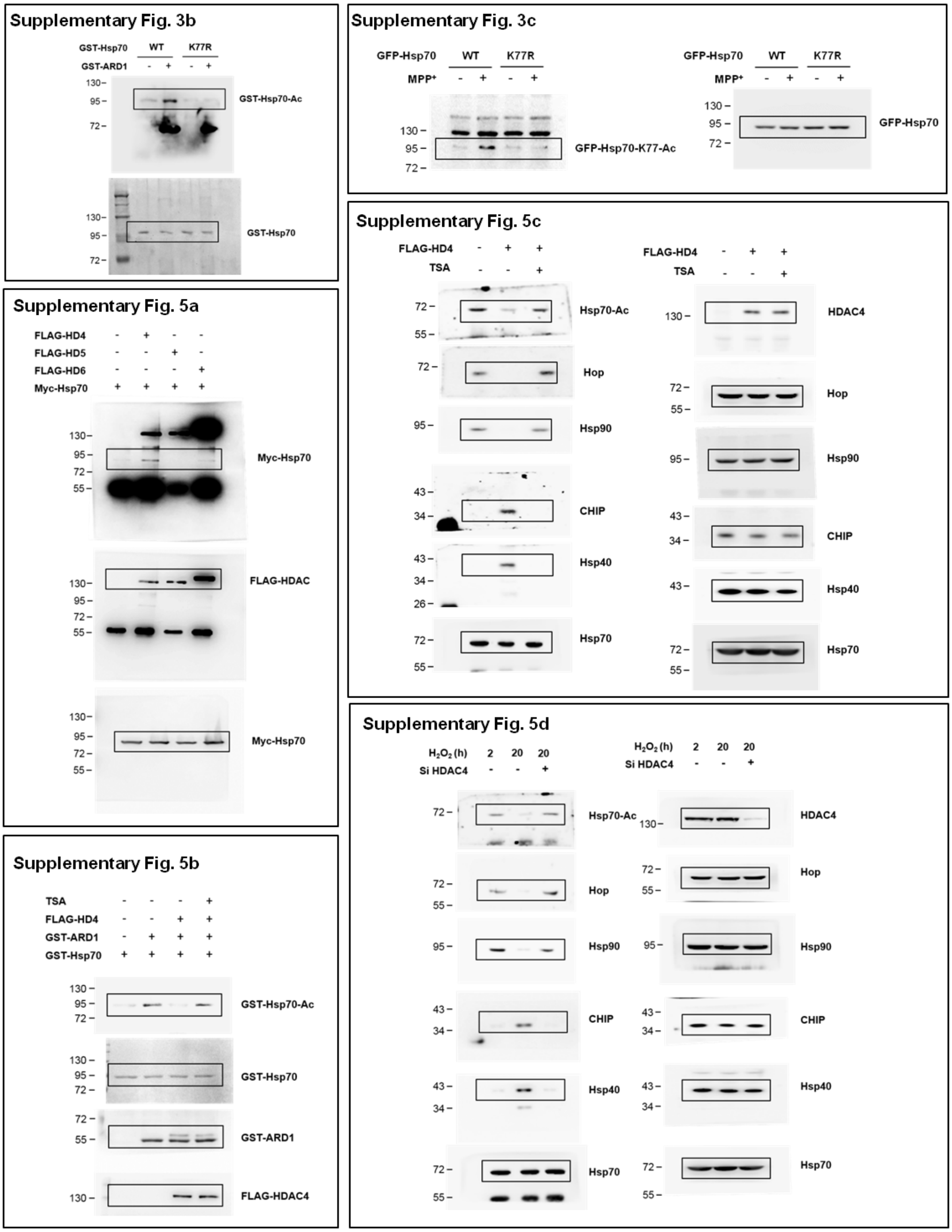

Supplementary Figure 12. Continued

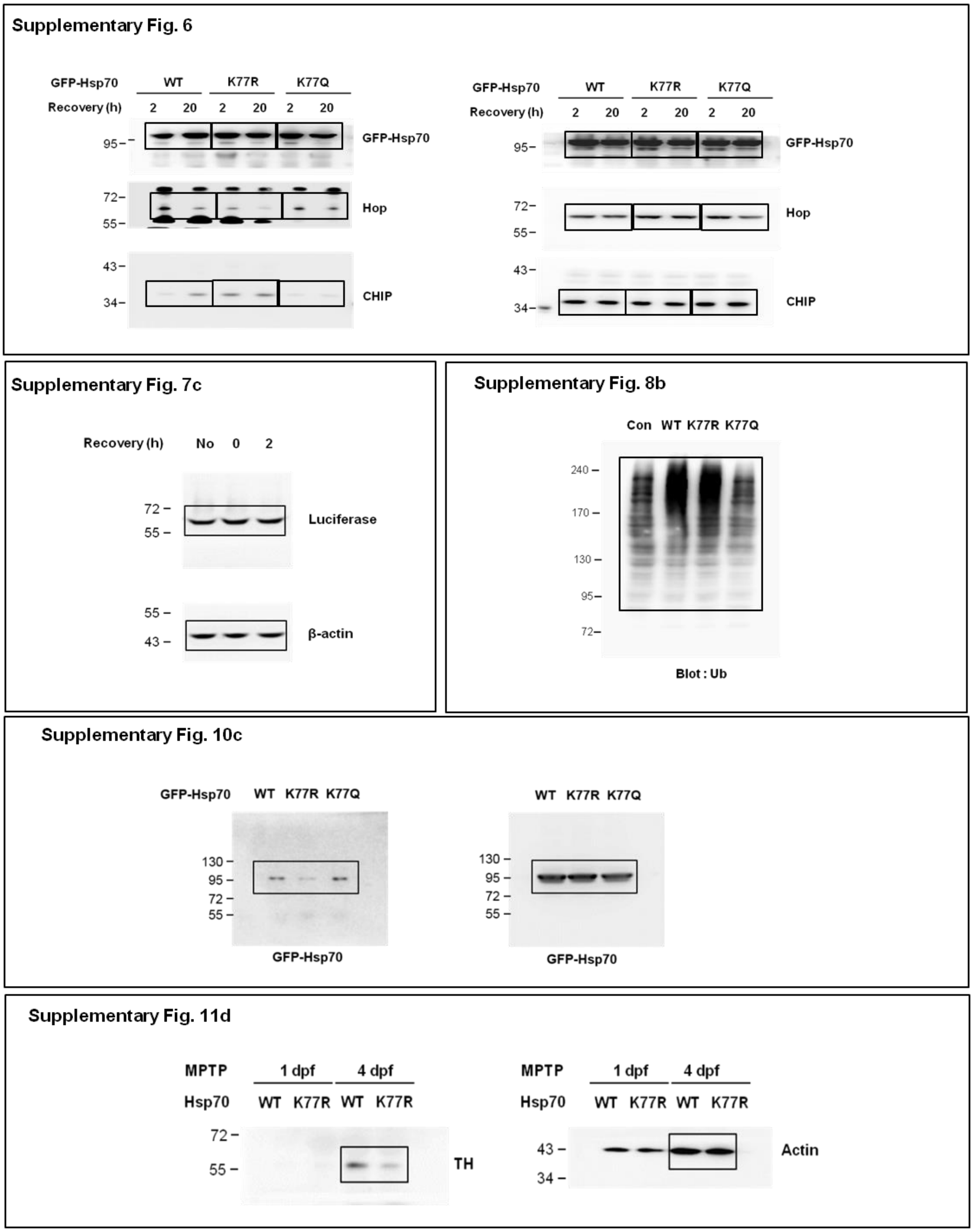

Supplementary Figure 12. Continued
